# Direct-Write Defects for Bias-Free Two-Dimensional Optical Synapses Towards In-Sensor Motion Detection

**DOI:** 10.1021/acs.nanolett.6c03873

**Published:** 2026-10-05

**Authors:** Tsz Wing Tang, Yunxia Hu, Yuxiang Li, Mohsen Tamtaji, Md Saif Hasan, Jiawen You, Jinghan Shen, Sheng Pei, Jun Wang, Yaxuan Li, GuanHua Chen, Zhaoli Gao, Nan Zhang, Zhengtang Luo

**Affiliations:** † Department of Chemical and Biological Engineering, William Mong Institute of Nano Science and Technology, and Hong Kong Branch of Chinese National Engineering Research Center for Tissue Restoration and Reconstruction, 58207 The Hong Kong University of Science and Technology, Hong Kong 999077, P. R. China; ‡ Beijing Engineering Research Center of Mixed Reality and Advanced Display, School of Optics and Photonics, 47833 Beijing Institute of Technology, Beijing 100081, P. R. China; § 645827 The Hong Kong Quantum AI Lab Limited, Hong Kong SAR 999077, P. R. China; ∥ Department of Chemistry, 25809 The University of Hong Kong, Hong Kong SAR 999077, P. R. China; ⊥ Department of Biomedical Engineering, 26451 The Chinese University of Hong Kong, Hong Kong 999077, P. R. China

**Keywords:** optical synapse, direct defect writing, transition metal dichalcogenides, homojunction, image recognition, motion trajectory prediction

## Abstract

Optoelectronic synaptic devices, particularly those based on two-dimensional (2D) transition metal dichalcogenides (TMDs), emulate biological synaptic functions, promising in neuromorphic computing. However, most TMD-based synapses rely on external gate or bias control, leading to complicated device architecture and high-power consumption. Here, we demonstrate a gate-free and bias-free optical synapses, based on monolayer molybdenum disulfide (MoS_2_) homojunction with its counterpart made of spatially controlled sulfur (S) vacancies, obtained through laser patterning. By precisely controlling the defect density from 0.15 nm^–2^ to 0.78 nm^–2^, we effectively modify the synaptic memory from a short retention time (∼125 s) to a long retention time (>450 s). We further demonstrate neuromorphic computing from image denoising and recognition to motion trajectory prediction for autonomous driving, highlighting the device’s applicability in on-chip visual preprocessing. This work shows great promise for neuromorphic computing by offering a scalable platform for energy-efficient optoelectronic neuromorphic technology.

## Graphical abstract

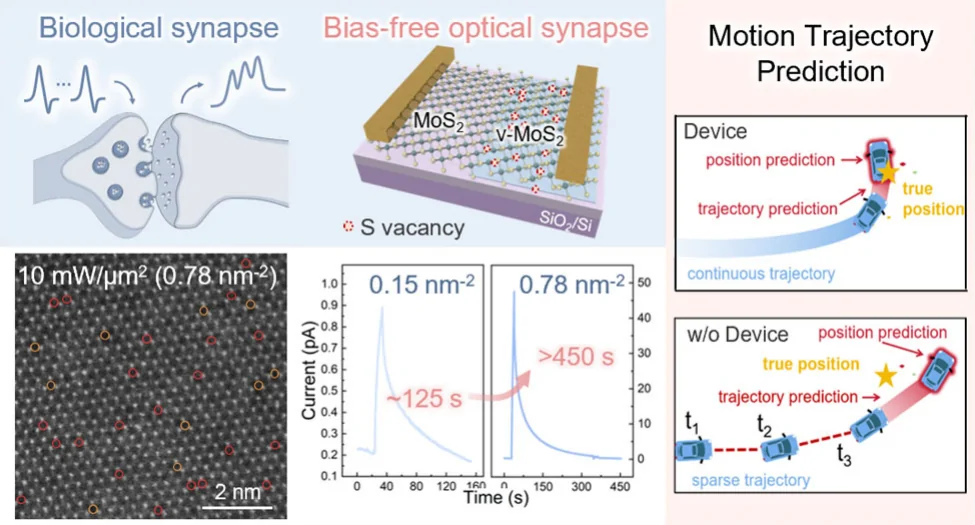

By merging processing and storage functions for higher data exchange efficiency and lower energy consumption, neuromorphic computing provides a solution to the problems of conventional von Neumann architecture computers.^1–3^ Resembling biological synapses, optoelectronic synaptic devices are a crucial aspect of artificial neural visual systems (ANVSs) and offer substantial potential for image preprocessing.^4–6^ These optical synapses respond to light stimuli by converting optical signals into postsynaptic currents. It simultaneously processes visual information at the pixel level and temporarily stores synaptic weights for a short time. These integrated functionalities enable both the efficient recording and temporal retention of visual information at the same time, which makes it possible to achieve the combination of photodetection and memory capabilities seamlessly.^7–9^

Two-dimensional (2D) transition-metal dichalcogenides (TMDs) are promising candidates for optoelectronic synaptic devices because of their unique properties, including distinctive atomic structure,^10,11^ adjustable bandgap,^12,13^ and excellent optical and electrical characteristics,^14^ etc.^15–17^ Efficient trapping sites in 2D TMDs to confine the photogenerated carriers have been shown to be an effective strategy to accomplish the optoelectronic synaptic function.^18–21^ In these cases, artificial intrinsic defective centers,^15^ gate-controlled design,^22,23^ or extrinsic allopatric trapping sites^8^ are frequently used to manipulate optoelectronic synaptic characteristics. However, the requirement for additional gate or bias voltages led to high energy consumption and also complicated the design and manufacture of device architectures. Material preparation for charge trapping functionalities also suffers from random defect distributions and concentrations, further necessitating external gate or bias voltage controls.^24^ Establishing gate- and bias-free optical synapses streamlines device architecture and improves energy efficiency by facilitating carrier segregation and transfer, thereby enabling autonomous optoelectronic systems.^25–27^

Here, we present a gate- and bias-free optical synapse by patterning sulfur (S) vacancies within molybdenum disulfide (MoS_2_) using direct laser writing. The spatially distributed S vacancies in MoS_2_ enable the formation of homojunction between pristine and vacancies-rich MoS_2_ regions, producing asymmetric Fermi level alignment. Additionally, it creates a discrepancy in the localization of the photogenerated carriers, resulting in a difference in Fermi levels at the junction upon light cessation. This configuration enables self-biased optical synapse without the need of external gate or source-drain voltages, and exhibits persistent postsynaptic current. Moreover, the synaptic plasticity from short retention time (∼125 s) to long retention time (>450 s) are achieved by precisely controlling the defect density from 0.15 nm^–2^ to 0.78 nm^–2^. Furthermore, neuromorphic computing from image denoising and recognition to motion trajectory prediction for autonomous driving has been achieved with increased accuracy, through the utilization of the excellent preprocessing capability of our optical synapse. This work establishes a versatile platform providing freedom for controlling optoelectronic synaptic performance, paving the way for scalable neuromorphic computing and advanced synaptic vision systems.

Spatial distributed S vacancies are introduced into the MoS_2_ lattice through 532 nm laser patterning (Figure 1a). More details are shown in Figures S1–S3. This vacancy patterning method is performed directly on as-grown MoS_2_ to construct homojunctions, eliminating the polymer-assisted transfer processes typically used in conventional fabrication methods, which often lead to residual contaminants. By precisely modulating the laser power density, the density of S vacancies in MoS_2_ is well-modified (Figure 1b). In addition to MoS_2_, this direct-written defect engineering strategy is extendable to other TMDs^28,29^ and van der Waals materials.^30^ This versatility makes the technique promising for tailoring electronic and optoelectronic properties across a broad family of multilayered 2D materials.^28^ While chalcogen vacancy generation via laser irradiation is a general phenomenon across the TMD family, processing parameters must be optimized to account for differences in defect formation energies and thermal stabilities. For instance, selenium (Se)-based TMDs generally form vacancies at lower laser powers than S-based TMDs owing to their lower thermal stability. Consequently, precise optimization of laser conditions is essential to reliably tailor the electronic and optoelectronic properties across diverse multilayered 2D materials.^31,32^ The densities of S vacancies, as quantitatively analyzed from scanning transmission electron microscopy (STEM), increase from 0.06 nm^–2^ in pristine MoS_2_ to 0.78 nm^–2^ in the 10 mW/μm^2^ laser-patterned MoS_2_, demonstrating precise control over defect concentrations (Figures 1c and S4). This modulation is essential for tailoring the photogenerated carrier trapping properties in MoS_2_ (Figure 1d).

**Figure 1:**
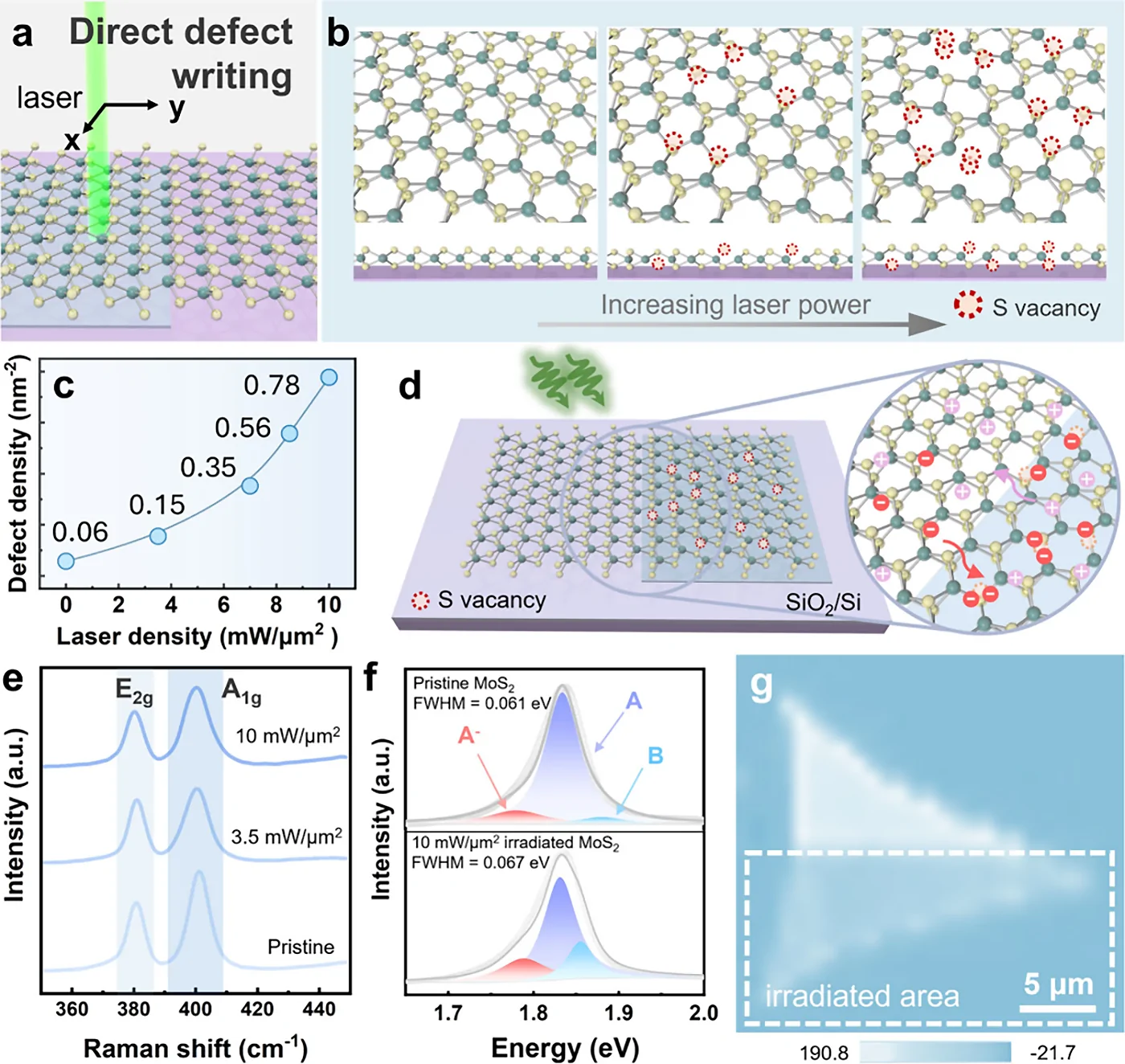
Spatially controlled S vacancies in MoS_2_ via direct laser writing. (a) Schematic illustration of direct defect writing for introducing S vacancies into MoS_2_. (b) Illustration of the correlation between patterning power densities and resulting S vacancies densities. (c) The correlation between laser power densities and S vacancies concentratons. (d) Photogenerated carrier migration dynamics with carrier trapping near S vacancies sites. (e) Raman spectra of pristine and 3.5, 10 mW/μm^2^ laser-irradiated MoS_2_, and (f) photoluminescence (PL) analysis for excitonic emissions of pristine MoS_2_ and 10 mW/μm^2^ laser-irradiated S vacancy MoS_2_. (g) Corresponding PL intensity mapping (at 1.825 eV) of the 10 mW/μm^2^ laser-patterned MoS_2_.

Figures 1e and S5 show the Raman and photoluminescent (PL) spectra of the pristine and S vacancy MoS_2_. In particular, the dominant Raman peaks for both pristine and S vacancies MoS_2_ are located at 384 and 404 cm^–1^, corresponding to the *E*_2g_ and *A*_1g_ vibrational modes of monolayer MoS_2_ with frequency difference approximately 20 cm^–1^.^33,34^ More details about the Raman and PL analysis are shown in Figures S6–S8. The PL spectra exhibit A and B exciton emissions from direct bandgap transitions, with A exciton corresponding to electron–hole pair recombination and B exciton arising from spin–orbit coupling-induced valence band splitting.^35–37^ The emission peaks of the A and B excitons in both pristine and S vacancy MoS_2_ are observed at approximately 1.83 and 1.87 eV, respectively, consistent with reported values (Figure 1f).^38–40^ However, the additional peak associated with negatively charged trion (A^–^) that consisting of an A exciton with one additional electron demonstrates an increased intensity after laser patterning, indicating the electrons doping resulting from S vacancies introduction.^36^ In addition, the PL intensity mapping images of the MoS_2_ homojunction are illustrated in Figures 1g and S7, wherein the laser-patterned region shows a uniform quenching of PL intensity. The detailed analysis of the PL spectra under different laser irradiation densities, focusing on the change in A, B, and A^–^ excitons, further confirms the controlled introduction of S vacancies (Figure S8).

The transmission electron microscopy (TEM) and STEM are employed to examine the structural evolution of MoS_2_ before and after laser irradiation to validate S vacancy introduction. A clear lattice pattern is observed in TEM image indicates that high-quality crystallinity maintains after laser patterning (Figure 2a). The *d*-spacing of approximately 0.27 nm corresponds to the 100 lattice planes. The selected area electron diffraction (SAED) pattern further indicates that the synthesized MoS_2_ is single crystalline. More details about TEM-energy dispersive X-ray spectroscopy (TEM-EDS) analysis are shown in Figure S9. The STEM images confirm precise control over the S vacancy density (Figures 2b,c). Two types of S vacancies are observed, monovacancies (single S atom missing) and di-S vacancies (both top and bottom S atoms missing) (Figure S10). Quantitative analysis reveals that the S vacancy density increases from 0.06 nm^–2^ in pristine MoS_2_ (6 S vacancies) to 0.78 nm^–2^ in the 10 mW/μm^2^ laser-irradiated MoS_2_ (22 S vacancies and 10 di-S vacancies). Moreover, both S vacancies and di-S vacancies act as effective electron trapping centers^41,42^

**Figure 2:**
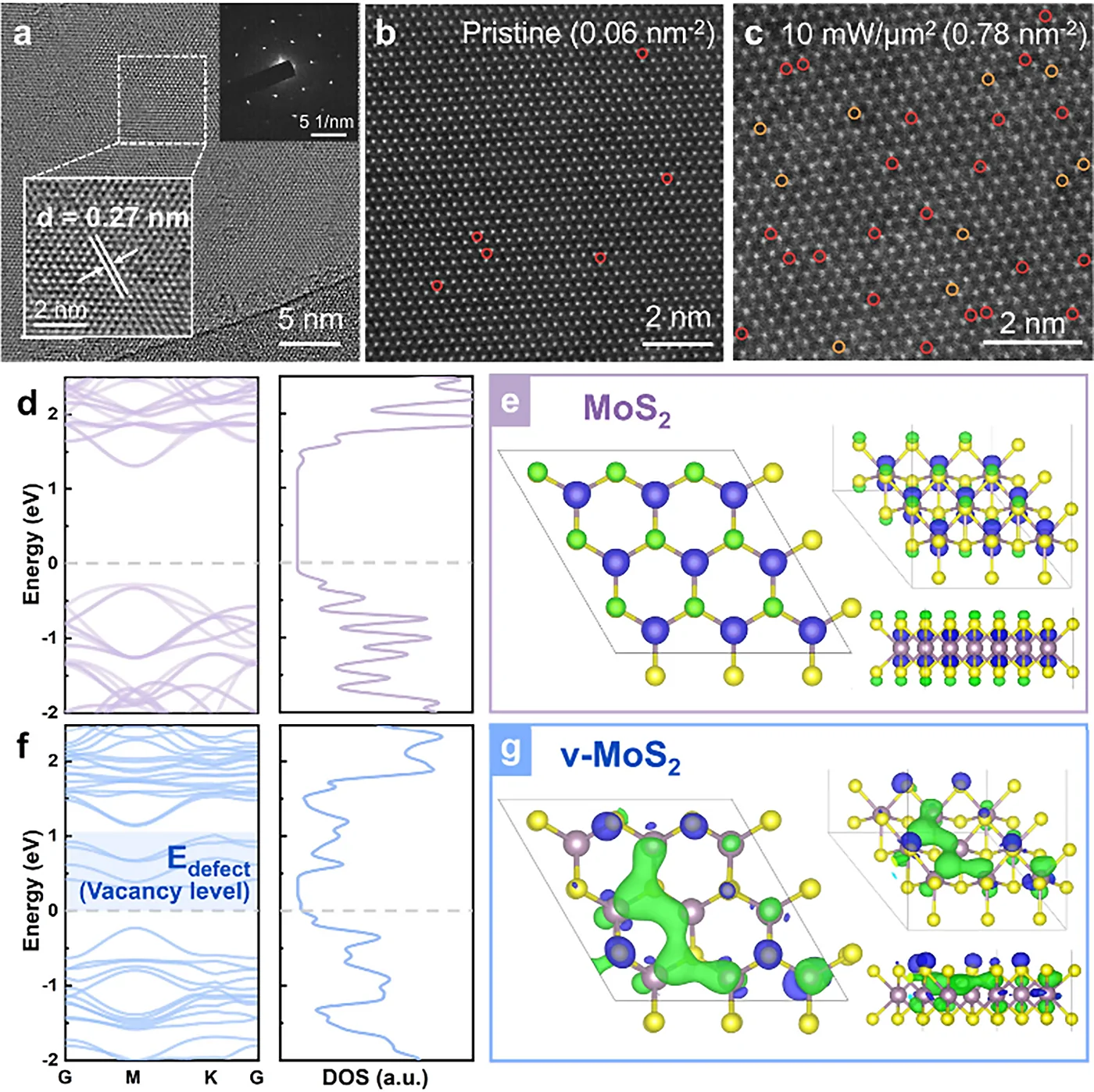
Carrier trapping at S vacancies in MoS_2_. (a) Transmission electron microscopy (TEM) image and corresponding selected-area electron diffraction (SAED) pattern (inset) of the 10 mW/μm^2^ laser-patterned MoS_2_. (b, c) Scanning TEM (STEM) image of (b) pristine and (c)­10 mW/μm^2^ laser-patterned MoS_2_ with introduced single S vacancies (highlighted in red) and double S vacancies (highlighted in orange). Density functional theory (DFT) calculated band structure and density of states (DOS) of (d) pristine MoS_2_ and (f) S vacancies MoS_2_. The Highest Occupied Molecular Orbital (HOMO) charge density for (e) pristine MoS_2_ and (g) S vacancies MoS_2_, shows the vacancies-induced asymmetric charge distribution (isosurface value = 3.6 e^–7^ e/Å^3^).

DFT calculations are performed to investigate the role of carrier trapping within S vacancy MoS_2_. With a quantified S vacancy density of only 0.06 nm^–2^ (Figures 2b and S4), the unirradiated MoS_2_ exhibits exceptionally high crystalline quality and negligible electronic structure perturbation (Figure S11). The calculated bandgap of pristine MoS_2_ is approximately 1.59 eV, consistent with previously reported values (Figure 2d).^43^ Furthermore, incorporating S vacancies into MoS_2_ disrupts the crystal lattice, introducing deep defect bands between the CB and VB. (Figure 2f). These defect-induced electronic states act as carrier trapping sites that localize photogenerated carriers and hinder electron–hole recombination, thereby prolonging carrier retention time.^44,45^ Highest occupied molecular orbital (HOMO) charge density for pristine (Figure 2e) and S vacancy MoS_2_ (Figure 2g) are calculated. This comparative analysis visually shows the redistribution of charge density caused by S vacancy introduction, revealing the region of localized electron availability and deficiency. For the pristine MoS_2_, the electron availability (green) and electron deficiency (blue) are evenly distributed due to a symmetric crystal lattice with a regular atomic arrangement and consistent interatomic forces. The removal of the S atom disrupts the local bonding environment, leaving behind dangling bonds on the adjacent Mo atoms that redistribute charges into localized electron-rich or -deficient regions, which serve as effective sites for localizing photogenerated carriers.

To investigate the charge trapping dynamics, Kelvin probe force microscopy (KPFM) is conducted on the pristine MoS_2_/0.78 nm^–2^ S vacancies MoS_2_ homojunction samples on SiO_2_/Si under three distinct conditions: in darkness, under light illumination, and after light cessation (Figure 3a–c). A gold (Au, 100 nm) pad functions as a reference standard for quantitative potential measurements. More importantly, substrate-induced baseline offsets were eliminated by extracting the relative Fermi level difference (Δ*E*_F_) across the shared substrate, revealing the intrinsic potential shift caused by S vacancies. Specifically, the difference in Fermi levels between Au and S vacancies MoS_2_ increases from 217 to 231 meV under light illumination, subsequently relaxes down to 227 meV after light cessation. Concurrently, the Fermi level difference between S vacancies MoS_2_ and pristine MoS_2_ rises from 149 to 173 meV under photoexcitation, then relaxes to 153 meV in postillumination. The KPFM image under light illumination shows that S vacancy MoS_2_ region exhibits an obviously increased surface potential compared to that in dark condition, confirming effective electron trapping at the S vacancy sites. More details about the KPFM analysis are shown in Figures S12 and S13.

**Figure 3:**
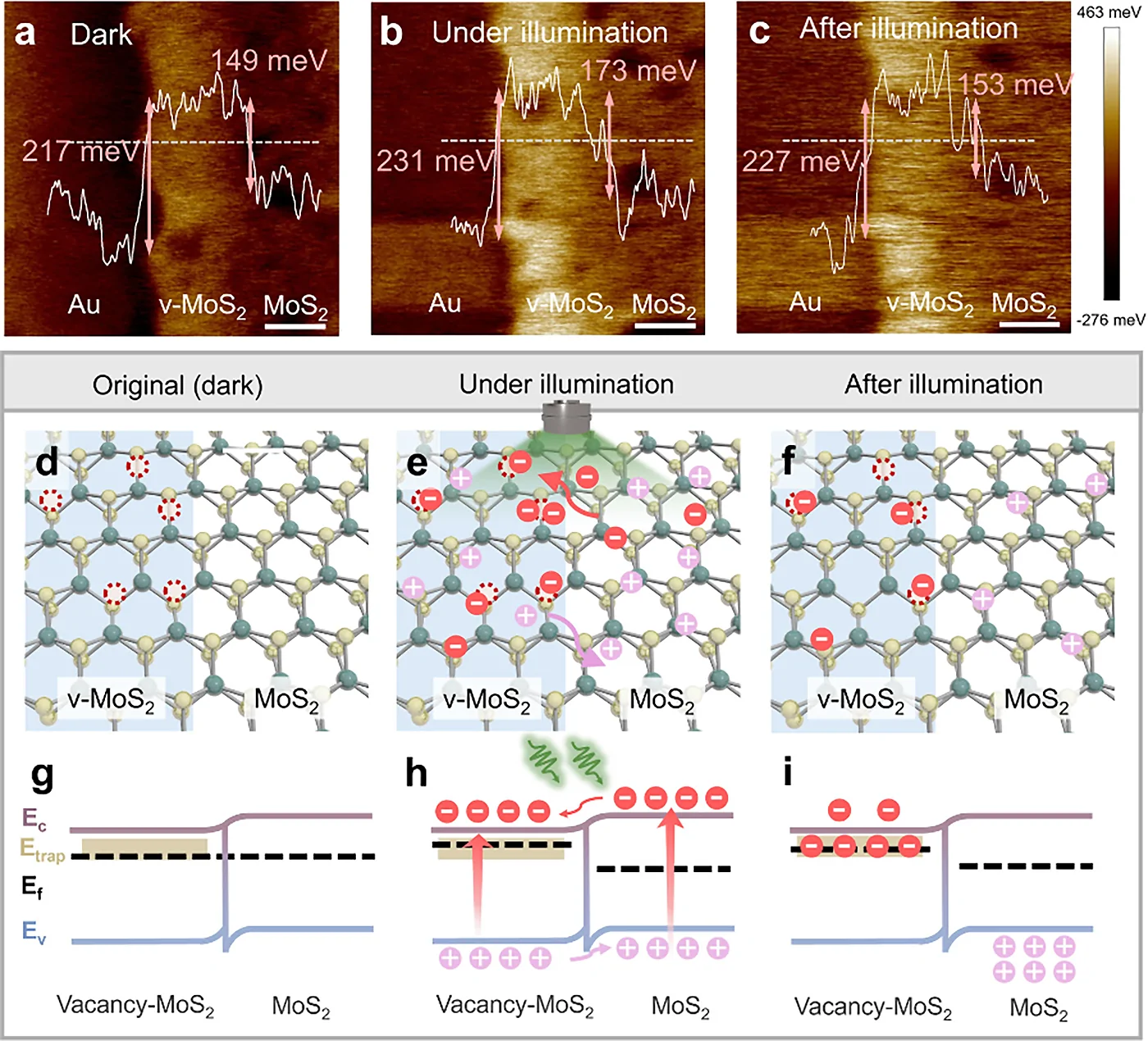
Surface potential evolution and charge trapping kinetics under optical stimulation. KPFM surface potential mapping of the gold (Au)/0.78 nm^–2^ laser-patterned MoS_2_/pristine MoS_2_ homojunction (scale bars are 1 μm, white dashed lines indicate surface potential profile extraction locations) (a) under dark condition, (b) under illumination (520 nm), and (c) after illumination. (d–f) The corresponding schematics of carrier generation, migration, and recombination processes and (g–i) band alignment diagrams under (d, g) dark conditions, (e, h) under illumination, and (f, i) after illumination, respectively.

Figure 3d–f depicts defect-induced photogenerated carrier trapping under both photoexcitation and postillumination states. The formation of a lateral homojunction between pristine MoS_2_ and S vacancy MoS_2_ creates a built-in electric field at the depletion region, due to their initial Fermi level difference.^45,46^ Under illumination, the photogenerated electrons and holes are separated by the electrical field. Crucially, electrons become selectively trapped at the localized trapping center of S vacancy MoS_2_, due to the deep defect states that were explained in the DFT session.^36^ The corresponding band alignments under different light conditions are illustrated in Figure 3g–i. The accumulation of negative charge at the S vacancy sites leads to an upward shift of the Fermi level in the S vacancy MoS_2_.^8^ In contrast, the photogenerated holes migrate to the pristine MoS_2_ region due to the built-in electric field, explaining the observed increase in Fermi level disparity at the junction. Trapped charges in deep defect states that are not immediately released upon light cessation result in slow electron–hole recombination.^45,47^ The gradual relaxation of the Fermi levels towards initial positions and the prolonged persistence of this nonequilibrium state explain the synaptic retention behavior without external bias.^30,48^

Figure 4a,b depicts the transmission of refined visual information from the retina to the visual cortex. As shown in Figure 4a, the human visual system integrates sensing and processing functions, beginning with the retina receiving light signals and converting them into physiological electrical signals within the synaptic network.^6,8,49^ In artificial synapses designed for neuromorphic computing, dynamic learning and memory functions are emulated through stimulus such as electrical or optical spikes, as illustrated in Figure 4b. The modulation of synaptic weight is an important feature of artificial synapses because it determines the duration of memory retention and controls the modification between short-term plasticity (STP) and long-term plasticity (LTP).

**Figure 4:**
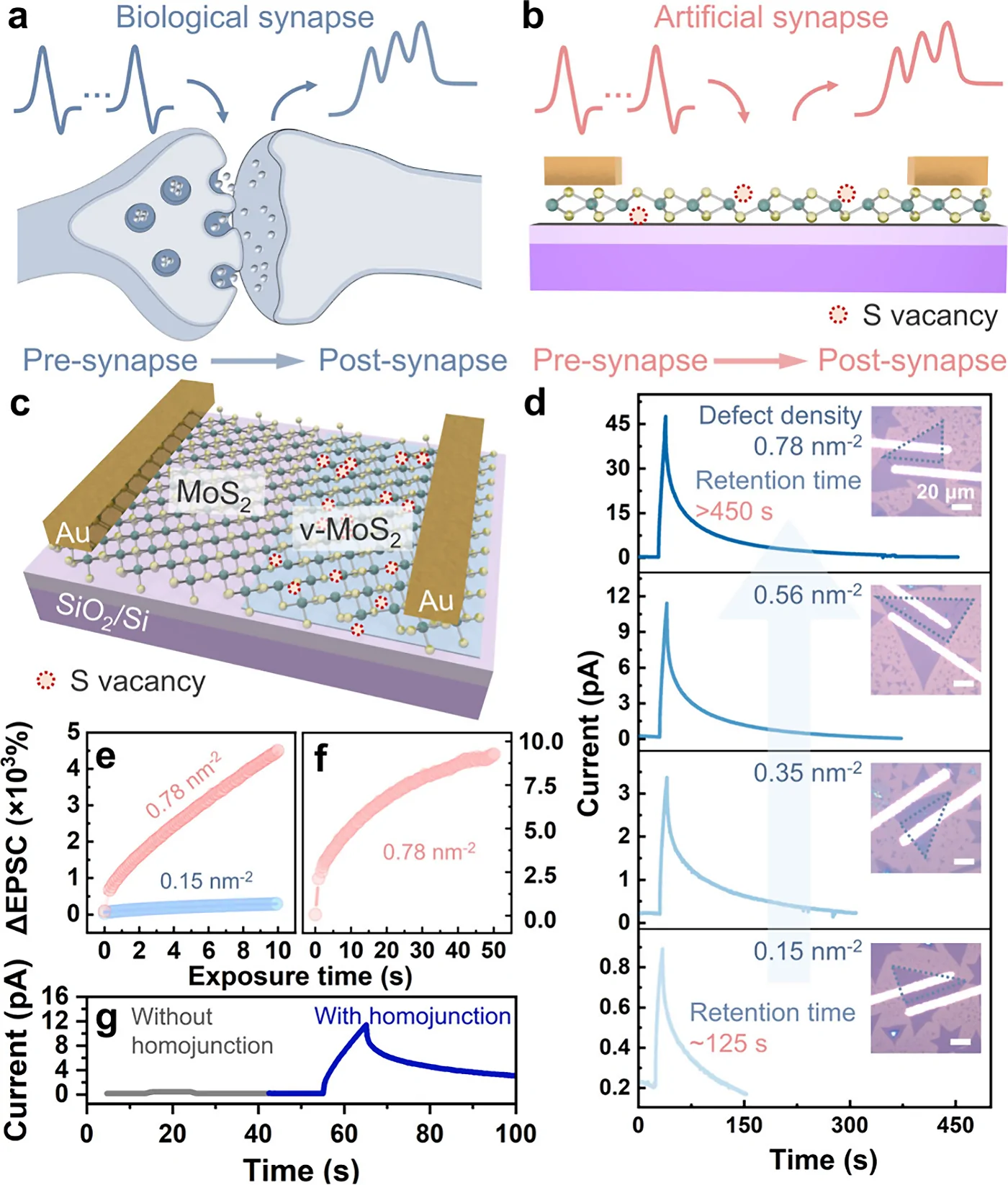
Bias-free optical synapses based on S vacancies MoS_2_/pristine MoS_2_ homojunctions with tunable synaptic weight. Schematic of (a) biological synapse in human visual system and (b) artificial synapses consisting of MoS_2_. (c) Device architecture of the self-biased pristine MoS_2_/ S vacancies MoS_2_. (d) Photoresponses of the laser irradiated MoS_2_ homojunctions with increasing densities of S vacancies from 0.15 nm^–2^ to 0.78 nm^–2^ under 10 s optical exposure (520 nm, 26.70 mW/cm^2^), insets are optical images of MoS_2_ devices with laser-patterned regions highlighted by dotted lines. Excitatory postsynaptic current (EPSC) enhancement of MoS_2_homojunction with S vacancies densities (e) 0.15 nm^–2^ and 0.78 nm^–2^ under 10 s exposure and (f) 0.78 nm^–2^ under 50 s exposure. (g) Photoresponse of devices without homojunction (entire channel uniformly patterned with 10 mW/μm^2^ laser irradiation) and with S vacancy MoS_2_/pristine MoS_2_ homojunction under zero-gate and -bias conditions.

To evaluate the gate-free and bias-free optical synaptic properties, a homojunction device consisting of S vacancies MoS_2_ and pristine MoS_2_ regions has been constructed (Figure 4c). Symmetric Au electrodes are employed to preclude the formation of an asymmetric contact-induced electric field. Consequently, the observed self-biased behavior is attributed to the built-in electric field at the homojunction rather than the asymmetric contact. More importantly, all postsynaptic current measurements are conducted under zero external gate and bias voltage (Figure S14). Therefore, the electrical energy consumption per synaptic event, calculated as *E* = *V* × *I* × *t*, is zero, demonstrating ultralow power consumption.

Through controlling the power density from 3.5 to 10 mW/μm^2^, the S vacancy density can be artificially modified from 0.15 nm^–2^ to 0.78 nm^–2^, enabling controlled transition of synapse weights from short retention time (∼125 s) to long retention time (>450 s) (Figure 4d). Under 10 s illumination, the postsynaptic current in the MoS_2_-based synaptic device exhibits a rapid increase due to the prompt generation and subsequent trapping of the photogenerated charge carriers. After the cessation of illumination, the current retains and goes through a slow decay process, which is governed by the slow release and recombination of the trapped charges. The self-biased synaptic properties are facilitated by the persistent unbalanced Fermi level alignment under the post-stimulation state.^15,25^ This alignment creates a discrepancy of the localization of the photogenerated carrier between the junction, thereby eliminating the need of external electrical fields to maintain the synaptic functionality.^50^ A higher density of S vacancies creates more availability of trapping sites and strengthens the built-in electric field, which lengthens the photogenerated carrier retention and prolongs postsynaptic current decay. More details about the device’s uniformity and stability are shown in Figures S15 and S16.

An excitatory postsynaptic current (EPSC) serves as a key quantitative indicator for identifying synaptic strength and plasticity in neuromorphic systems. The change in EPSC (ΔEPSC) is calculated as the normalized percentage change in the postsynaptic current amplitude relative to its dark value. The defect density-dependent ΔEPSC shows the increase of excitatory current response with higher defect density (Figure 4e). The enhanced ΔEPSC in S vacancies MoS_2_ is primarily due to a greater density of photogenerated carriers, driven by improved charge trapping capability and enhanced charge separation from the significant difference in Fermi levels between the pristine MoS_2_ and S vacancies MoS_2_.^25,51^ The ΔEPSC increases with longer exposure time due to a higher concentration of the photogenerated carriers, resulting in a larger postsynaptic current (Figure 4f). To confirm the critical role of spatially controlled defect engineering for self-biased optical synapses, the photoresponse of the device without homojunction has been characterized, exhibiting no retention characteristics under zero bias (Figure 4g). This sharply contrasts with the prolonged retention observed in the homojunction MoS_2_, demonstrating that the strategy of spatially patterned S vacancies in MoS_2_ is critical for the operation of self-biased optical synapses under gate-free and bias-free conditions.

The exposure time-dependent photoresponse is shown in Figure S17. In addition, the device also exhibits excellent spike-number-dependent plasticity (SNDP) behavior, characterized by a systematic increase in photoresponse magnitude with an increased number of optical pulses. Repeated optical stimulation induces cycle-to-cycle potentiation in the MoS_2_ homojunction as cumulative carriers trapping at S vacancies progressively enhances the postsynaptic current. Figure 5a–c compares the SNDP results under different numbers of light pulse stimuli, where the postsynaptic current amplitude shows a significant reinforcement with increasing number of pulses. Paired-pulse facilitation (PPF) is a fundamental form of STP, where the second synaptic response is temporarily enhanced when two closely spaced pulses are applied. The calculated PPF index (*A*_2_/*A*_1_ ratio) is 1.14 (Figure 5a). This result demonstrates a strong synaptic weight modulation that mimics biological STP. The variation of synaptic weight, quantified by the ratio *A*_*n*_/*A*_1_, represents the amplification of the postsynaptic current after *n* optical pulses (*A*_*n*_) relative to the response after the first pulse (*A*_1_) and indicates the strength of plasticity and reflects the ability to integrate the sequential stimuli. A significant synaptic amplification ratio of *A*_50_/*A*_1_ > 3.1 is exhibited, demonstrating the efficient optically driven synaptic behavior (Figure 5d). This high synaptic amplification highlights the robustness of the artificial synapse, enabling adaptive capability in neuromorphic computing with high fidelity. More importantly, the synaptic photoresponse characteristics showcase excellent bioinspired neuromorphic functionalities, including synaptic plasticity, exposure time-dependent photoresponse, and SNDP, confirming that the defect-engineered MoS_2_ homojunction effectively emulates key biological learning rules. In addition, our self-biased optical synapse serves as a building block for neuromorphic in-sensor detection and artificial visual neural networks, where ultralow energy consumption and zero external voltage provide valuable advantages.

**Figure 5:**
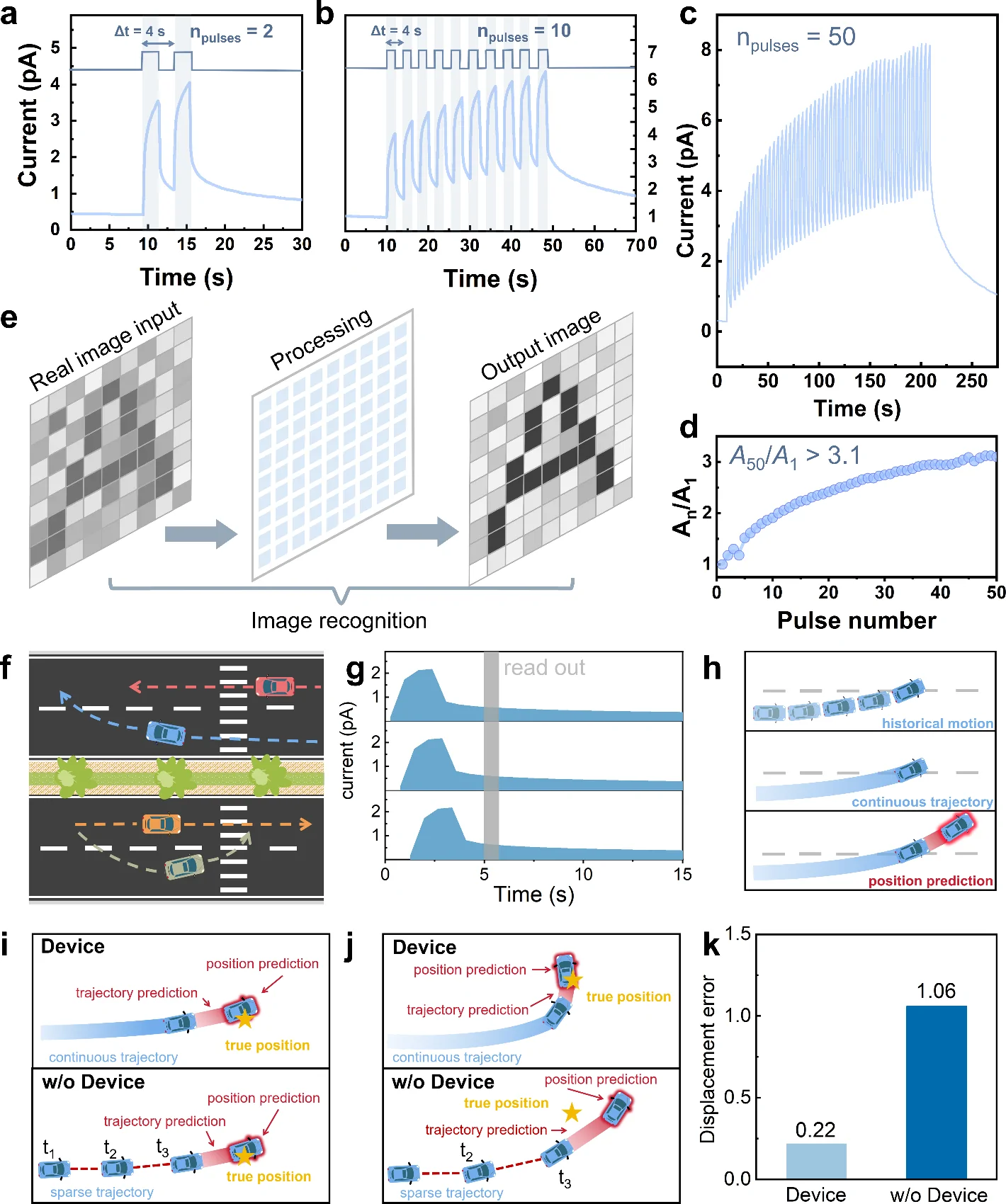
Bioinspired neuromorphic functionality. (a) Spike-number-dependent plasticity (SNDP) of the 0.78 nm^–2^ S vacancies MoS_2_/pristine MoS_2_ homojunction device under different pulse number *n*_pulse_ = (a) 2, (b) 10, (c) 50 (pulse width = 2 s, interpulse interval = 2 s). (d) Synaptic amplification ratio (*A*_n_/*A*_1_) as a function of pulse number. (e) Schematic illustrations of the neuromorphic image preprocessing and (f) vehicle trajectory prediction for autonomous driving and path planning. (g) The optical synapses in the array exhibit memory characteristics. (h) Moving objects sequentially activate the optical synapses in the array. (i) Target position prediction under small motion variations for systems with and without optical synapses. (j) Target position prediction under large motion variations, comparing systems with and without optical synapses against ground-truth positions. (k) Comparison of displacement errors in position prediction with and without optical synapses.

The inherent synaptic photoresponse characteristics of the gate-free and bias-free optical synaptic devices are utilized to perform neuromorphic image denoising preprocessing and recognition (Figure 5e). By exploiting the tunable persistent photoconductivity, noise suppression through reconfiguration of the image grayscale values is demonstrated (Figures S18–S22). This implementation not only validates the device’s functions for visual data preprocessing but also showcases potential for robust neuromorphic computing.

In dynamic traffic scenarios, the capability of perceiving future motion trends of surrounding objects is directly related to the decision-making safety of autonomous driving systems (Figure 5f). Constrained by discrete sampling and limited frame rates, conventional snapshot-based vision systems acquire only sparse historical position data that fails to preserve continuous temporal dynamics, thereby limiting the accuracy of downstream trajectory prediction. As shown in Figure 5g, when stimuli are applied at different times and read out simultaneously, the photocurrent response of the synapse can reflect the temporal order of the stimuli. When extended into a spatial array, optical synapses with temporal memory effects can continuously sense and record the motion process of dynamic targets (Figure 5h). Sequential pixel activation across the array directly encodes spatial positions and temporal motion sequences across the array, leveraging the device’s intrinsic memory characteristics to fuse trajectory-related spatiotemporal information.

On the basis of the continuously recorded trajectory information, future position prediction is further investigated. While conventional frame-based tracking suffers from prediction deviations and higher-order overfitting due to sparse historical trajectory data, the optical synapse-based method leverages high-density trajectory information to achieve stable, higher order motion fitting. The prediction error continuously decreased and eventually achieved the best performance with fifth-order model, the optimal results of both methods were adopted for evaluation (Table S1). Figure 5i,j presents the prediction results of the two methods under different motion trajectories. For trajectories with relatively small variations, both methods produced predicted positions close to the ground truth. However, for larger motion variations, the average position prediction error reduces from 1.06 to 0.22 after introducing memory-enabled optical synapse, (approximately 5 times improvement in prediction accuracy, Figure 5k). These results demonstrate that the proposed device effectively preserves continuous motion information and significantly improves trajectory prediction in complex dynamic scenarios, highlighting its potential for high-performance perception hardware in autonomous driving and edge intelligence.

In conclusion, we demonstrate a gate-free, bias-free optical synapse utilizing a homojunction incorporating regions of pristine and S vacancies MoS_2_. Spatial S vacancies in the MoS_2_ homojunction induce asymmetric carrier localization and unequal postillumination Fermi levels, enabling persistent postsynaptic current retention for self-biased, gate-free optical synapse operation. By modifying the defect density from 0.15 nm^–2^ to 0.78 nm^–2^, the tunable synaptic plasticity from short retention time to long retention time (∼125 s to >450 s) is achieved. We further validate the device’s neuromorphic functionality from image recognition to motion trajectory prediction for autonomous driving, aided by the excellent preprocessing capability of our optical synaptic device. This work demonstrates a new operational efficacy for in-sensor visual data preprocessing and opening new possibilities for neuromorphic computing.

## Supplementary Material



## Notes

Experimental section/methods, additional information, figures and table, including CVD setup, optical image, STEM images, Raman and PL analysis, TEM-EDS mapping images, STEM line-profile analysis, DFT-optimized ground-state atomic structures, KPFM line-profile analysis, illustration diagram of device measurements, device-to-device photoresponse, Long-term device stability, exposure time-dependent photoresponse, schematic of the three-layer neural network, image recognition results, and information on polynomial fitting-based trajectory prediction (PDF)
