# Supplementary material for: Direct-Write Defects for Bias-Free Two-Dimensional Optical Synapses Towards In-Sensor Motion Detection
Source: Nano Lett. 2026 Oct 5;26(40):14030–9. doi: 10.1021/acs.nanolett.6c03873 (PMC13674334; doi:10.1021/acs.nanolett.6c03873)
Supplement: Supplementary file 1 [file nl6c03873_si_001.pdf]

Supplementary Information

# Direct-Write Defects for Bias-Free Two-Dimensional Optical Synapses towards In-Sensor Motion Detection

Tsz Wing Tang[a#], Yunxia Hu[a#], Yuxiang Li[b], Mohsen Tamtaji[a,c], Md Saif Hasan[a], Jiawen You[a], Jinghan Shen[a], Sheng Pei[a], Jun Wang[a], Yaxuan Li[a], GuanHua Chen[c,d], Zhaoli Gao[e], Nan Zhang[b], Zhengtang Luo[a]*

[a]*Department of Chemical and Biological Engineering, William Mong Institute of Nano Science and Technology, and Hong Kong Branch of Chinese National Engineering Research Center for Tissue Restoration and Reconstruction, The Hong Kong University of Science and Technology, Hong Kong 999077, P. R. China;*

[b]*Beijing Engineering Research Center of Mixed Reality and Advanced Display, School of Optics and Photonics, Beijing Institute of Technology, Beijing 100081, China;*

[c] *The Hong Kong Quantum AI Lab Limited, Hong Kong SAR 999077, China;*

[d] *Department of Chemistry, The University of Hong Kong, Hong Kong SAR 999077, China;*

[e] *Department of Biomedical Engineering, The Chinese University of Hong Kong, Hong Kong 999077, P. R. China*

[#]*These authors contributed equally to this work.*

*E-mail: kezltuo@ust.hk.

## Experimental Section/Methods

### Material Preparation

The synthesis of monolayer $MoS_2$ on a silicon wafer ($SiO_2$/Si, 1 cm × 1 cm) was carried out using a CVD method, with ammonium molybdate tetrahydrate (AHM, Aladdin cas: 12054-85-2) and S powder serving as the precursors for Mo and S, respectively. A drop of diluted AHM solution (0.25 mM, 0.5 mL) was dried on the $SiO_2$/Si substrate and placed in the downstream region, while S powder (200 mg) was placed in the upstream. Before the growth, the quartz tube was cleaned by 200 sccm Ar flow for 15 minutes. Then, the substrate was heated to 790°C in 20 minutes ramping time. After the substrate reached the targeted temperature, the S powder was heated to around 350°C. The growth duration was 10 minutes; the tube furnace was then stopped after the growth and cooled rapidly to room temperature. The laser irradiation was conducted using a inVia Qontor (Renishaw Ltd.) spectrometer system with a 532 nm laser and tunable irradiation power. Controllable S vacancy introduction was achieved by systematically tuning the incident laser power density (3.5-10 mW/$\mu m^2$) while maintaining fixed beam spot size (1 μm), exposure time (1s) and pass count (single pass). To prevent material damage and ensure precise control over laser power density, all power levels used during the patterning process were calibrated using a power meter (LASERCHECK, Coherent, Serial No. 0610D24R).

### Characterization

The optical microscope characterization of the $MoS_2$ was conducted using a LEICA DMLM. The spectroscopic characterizations, including PL and Raman, of the as-grown $MoS_2$ were measured using a inVia Qontor (Renishaw Ltd.) spectrometer system with a 532 nm laser. The thickness of the as-grown sample was characterized by AFM (Dimension Icon, Bruker). KPFM measurements were conducted under ambient conditions using the same AFM system equipped with a Kelvin probe, with the sample electrically grounded to prevent floating charge accumulation. Before KPFM measurements, the samples were annealed at 150°C for 2 hours in tube furnace under 10:1 Ar:$H_2$ atmosphere to remove polymer residues, a common procedure to desorb contaminants and improve surface cleanliness. The as-grown $MoS_2$ was transferred onto a copper grid using PMMA-assisted transfer for TEM and STEM characterizations, conducted with a JEM 2010F (JEOL) under 60 kV.

### Device Measurement

The optoelectronic characterizations of S vacancies $MoS_2$/pristine $MoS_2$ homojunction optical synapse devices were conducted using a Keithley 4200 SCS semiconductor parameter analyzer and a FormFactor MPS 150 probe station. For optical stimulation, the device was illuminated with a 520 nm light source (MW-GX-520) positioned 20 cm above the sample at an intensity of 26.70 mW/cm$^2$. The light intensity was calibrated using a Vega Color Laser Power & Energy Meter.

**DFT Calculation**

The calculations were performed using the Vienna ab initio Simulation Package (VASP 6.1.0). The structures, band structures, density of states (DOS) of pristine $MoS_2$ and S vacancies $MoS_2$ were calculated using DFT with the Perdew–Burke–Ernzerhof functional and a plane-wave cutoff energy of 520 eV. A 6 × 6 × 1 k-point grid was used for structural relaxation in the Brillouin zone, following the Monkhorst-Pack scheme, with convergence criteria set to $10^{-6}$ eV for energy and 0.025 eV/Å for atomic forces. The DFT-D3 method was applied to account for the van der Waals interactions in both pristine $MoS_2$ and S vacancies $MoS_2$. For the DOS and energy band calculations, a 12 × 12 × 1 k-point grid was used, with a convergence criterion of $10^{-7}$ eV.

**Neural Network for Image Processing**

In the image processing stage, a pre-modeled synaptic response is introduced to enhance the contrast of the input image, aiming to simulate the perceptual enhancement mechanism of biological synapses. This process not only improves image contrast but also suppresses blur and enhances local detail information, thereby increasing the separability between the license plate region and the background. The Canny operator is then applied for edge detection to further highlight the structural features of the license plate. For license plate localization, candidate regions are extracted based on plate-specific features, followed by morphological operations to achieve accurate localization and segmentation. In addition, the Radon transform is employed to correct the tilt of the detected license plate. During character segmentation, the localized license plate image is binarized, and a column projection method is adopted to segment individual characters. The segmented characters are subsequently normalized to a size of 12×24 pixels. For character recognition, a three-layer BP neural network is constructed for classification. The input layer consists of 288 features, the hidden layer contains 30 neurons, and the output layer has 49 neurons corresponding to different character classes. The network parameters are randomly initialized and optimized using the conjugate gradient method (fmincg) with a maximum of 500 iterations and a regularization parameter $\lambda = 1$. Recognition results are obtained through forward

propagation.

**Motion Prediction Dataset**

To evaluate the trajectory prediction performance, a motion trajectory dataset was constructed based on the Clothoid curve model. Specifically, the trajectory length and terminal curvature were randomly generated, where the trajectory length ranged from 10 to 20 and the terminal curvature varied from −0.3 to 0.3. The initial curvature was fixed at zero, and the starting position was set to (0,0). Subsequently, smooth two-dimensional trajectories with continuous curvature variation were obtained by integrating the curvature function, followed by uniform sampling along the trajectory at fixed intervals. In total, 100 trajectories with different motion patterns were generated for experimental evaluation, and the Euclidean distance between the predicted and ground-truth positions was adopted as the metric for prediction error.

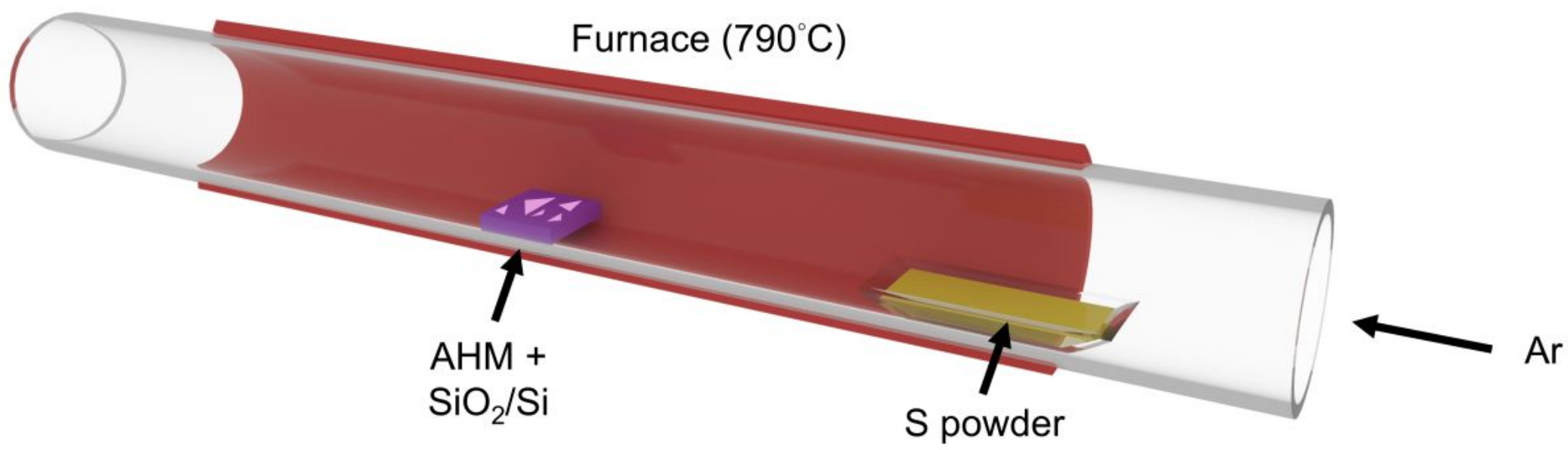


**Figure S1.** Illustration of the chemical vapor deposition (CVD) setup for the synthesis of monolayer $MoS_2$.

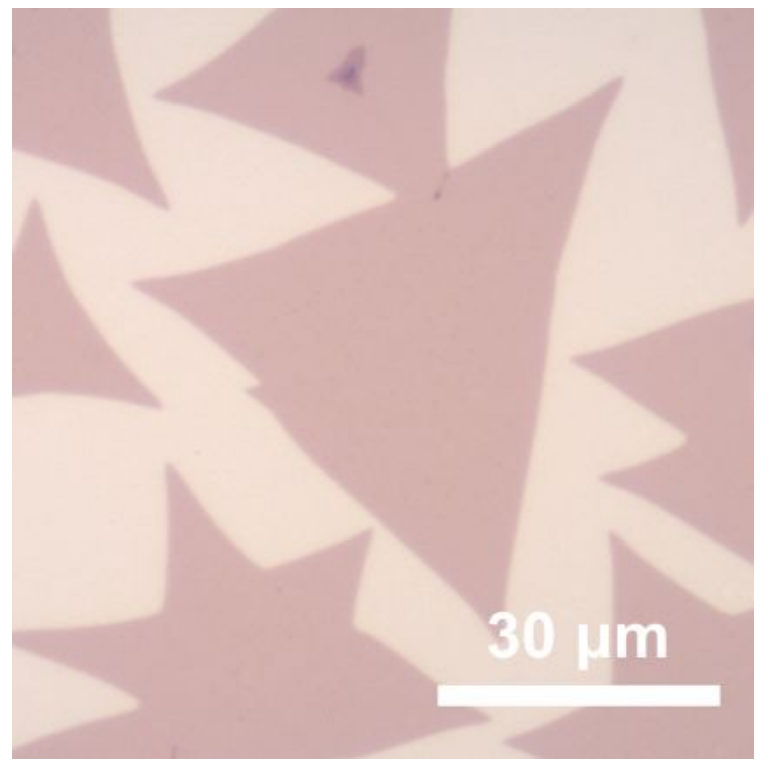


**Figure S2.** The optical image of the CVD grown monolayer $MoS_2$ on the $SiO_2$/Si substrate.

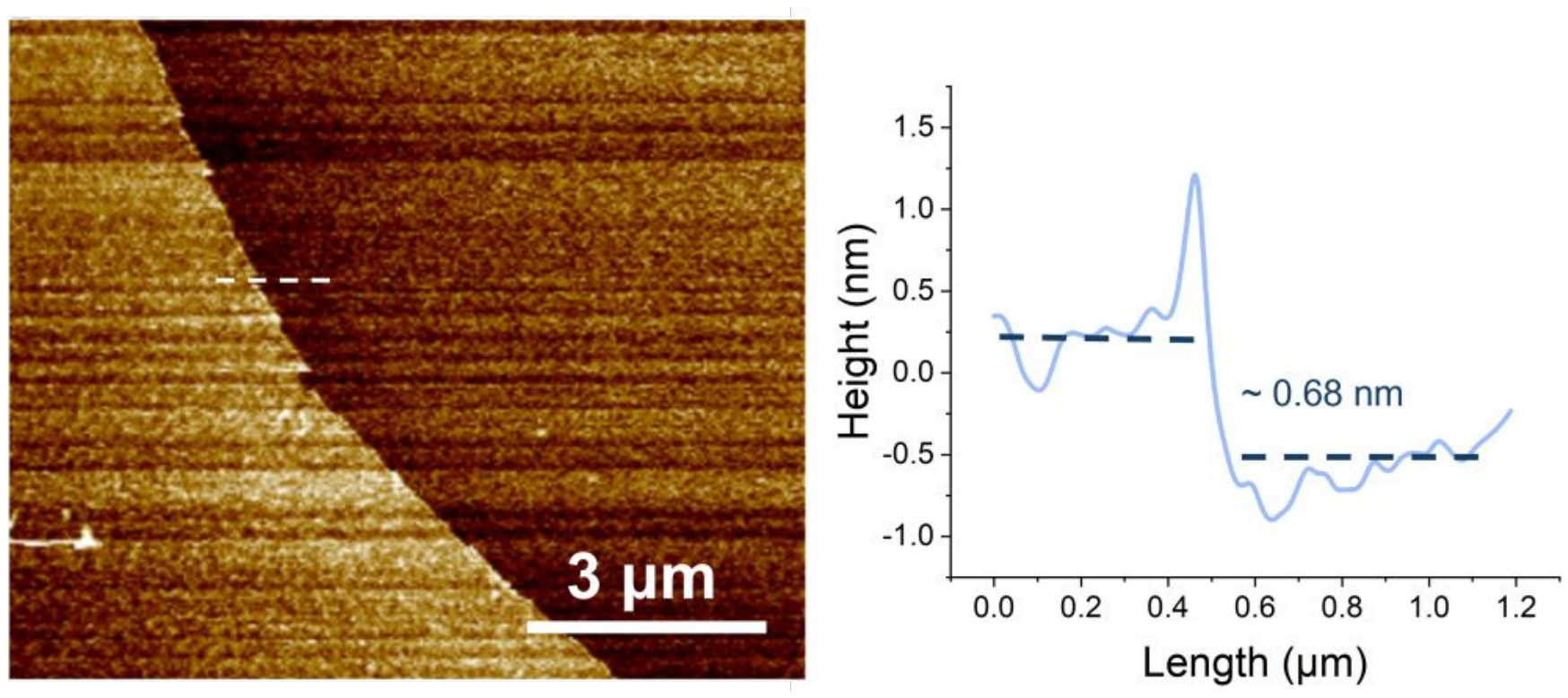


**Figure S3.** AFM image and the corresponding step height of CVD-grown $MoS_2$ with a measured thickness of ~ 0.68 nm. The thickness of ~ 0.68 nm evidence the monolayer feature of the CVD-grown $MoS_2$.

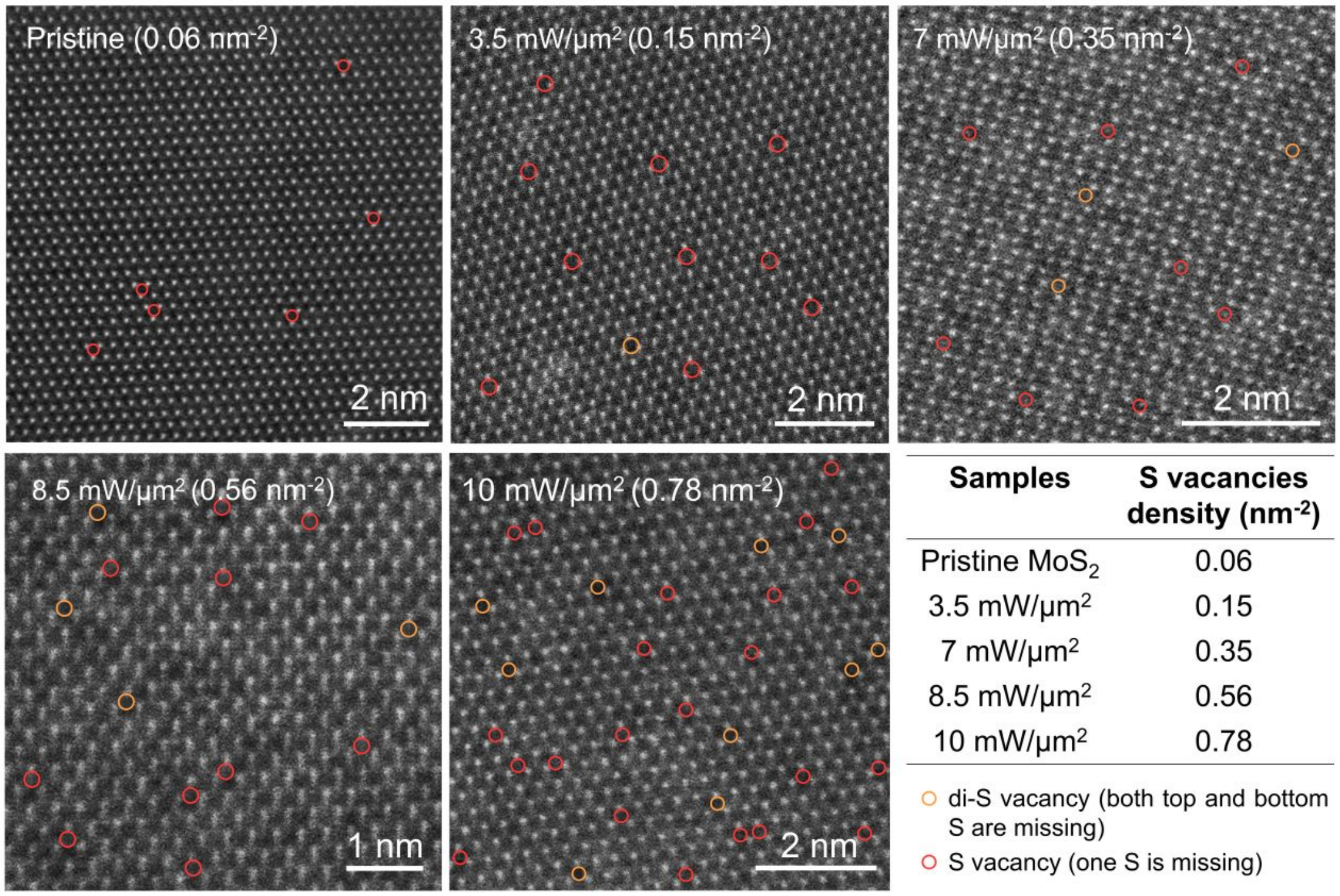


| Samples | S vacancies density ($nm^{-2}$) |
|---|---|
| Pristine $MoS_2$ | 0.06 |
| 3.5 mW/μ$m^2$ | 0.15 |
| 7 mW/μ$m^2$ | 0.35 |
| 8.5 mW/μ$m^2$ | 0.56 |
| 10 mW/μ$m^2$ | 0.78 |

**Figure S4.** Scanning transmission electron microscopy (STEM) is employed to identify the changes in S vacancies density between pristine $MoS_2$ and the samples patterned with power densities ranging from 3.5 to 10 mW/μ$m^2$. The STEM images of the $MoS_2$ sample and the corresponding quantitative analysis reveal that the total S vacancy density increases from 0.06 $nm^{-2}$ in pristine $MoS_2$ (6 S vacancies) to 0.78 $nm^{-2}$ in the 10 mW/μ$m^2$ laser-irradiated $MoS_2$ (22 S vacancies and 10 di-S vacancies). The creation of S vacancies typically involves the breaking of Mo–S bonds and the subsequent desorption of S atoms from the lattice. A single S vacancy requires the removal of one S atom, which can be achieved with relatively lower energy input (low-power laser patterning). In contrast, the formation of a double S vacancy requires higher energy to remove two S atoms, as this involves breaking additional Mo–S bonds and overcoming local lattice strain. However, the formation of a first single S vacancy weakens the local lattice, thereby facilitating the removal of an adjacent S atom to form a double S vacancy.[1,2] Therefore, the densities of double S vacancies increase when the laser power densities increase.

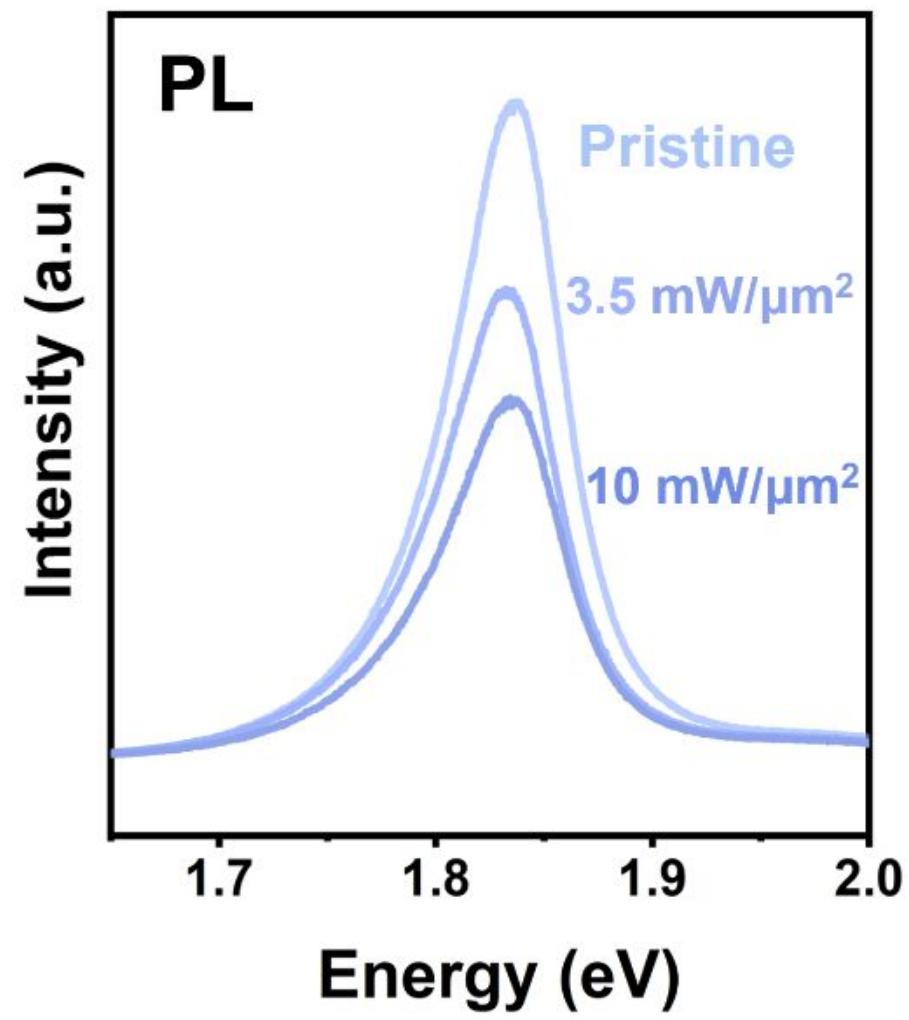


**Figure S5.** Photoluminescence (PL) spectra of pristine monolayer $MoS_2$ and S vacancies $MoS_2$ irradiated at 3.5 and 10 mW/μm$^2$ laser density, respectively.

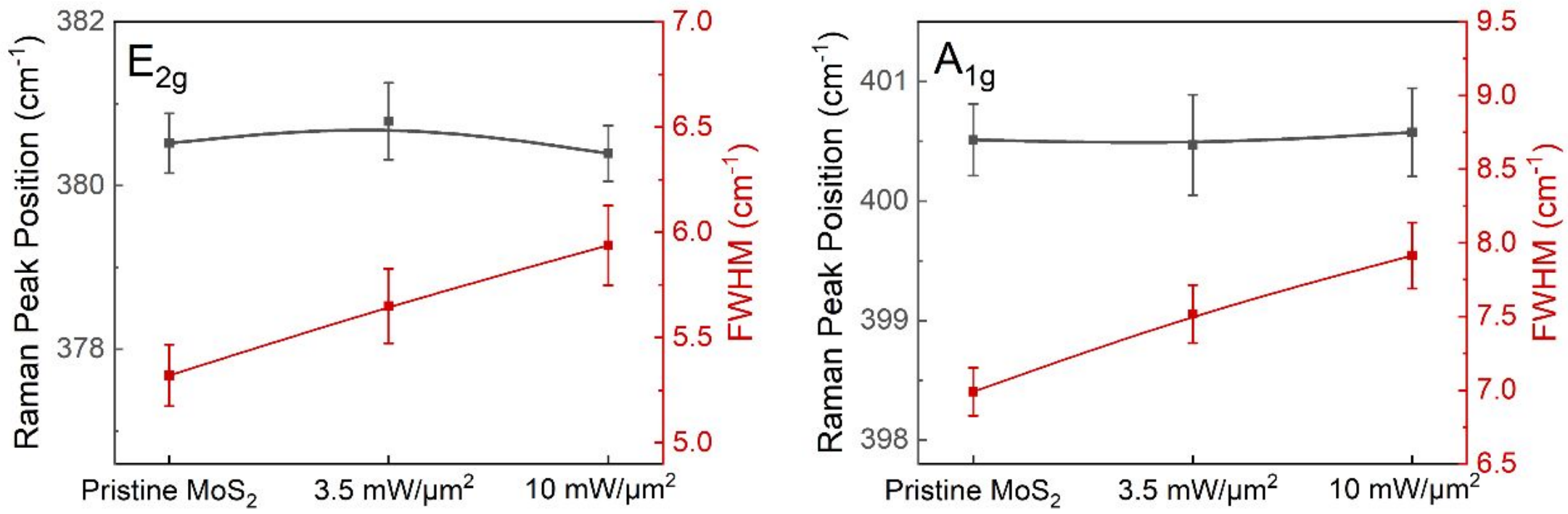


**Figure S6.** Raman peak position and full width at half maximum (FWHM) analysis of $E_{2g}$ and $A_{1g}$ vibrational modes for the $MoS_2$ under pristine condition and after different laser intensities irradiation (3.5 and 10 mW/μm$^2$). No significant shifts are observed in the Raman peak positions of either the $A_{1g}$ or $E_{2g}$ modes of monolayer $MoS_2$ following laser irradiation, indicating that the vibrational modes remain unchanged despite the introduction of S vacancies. However, there is a significant change in FWHM after the introduction of S vacancies in $MoS_2$. This change can be attributed to the decrease in crystallinity and the additional disorder and structural perturbations caused by the vacancy introduction, resulting in a broader distribution of vibrational frequencies and FWHM. It is worth noting that partial oxygen substitution or adsorption may occur at S-vacancy sites in CVD-grown $MoS_2$ and after laser treatment,[2-4] but only at a minimal level, and the samples are stored in a vacuum to prevent material passivation by oxygen and moisture.

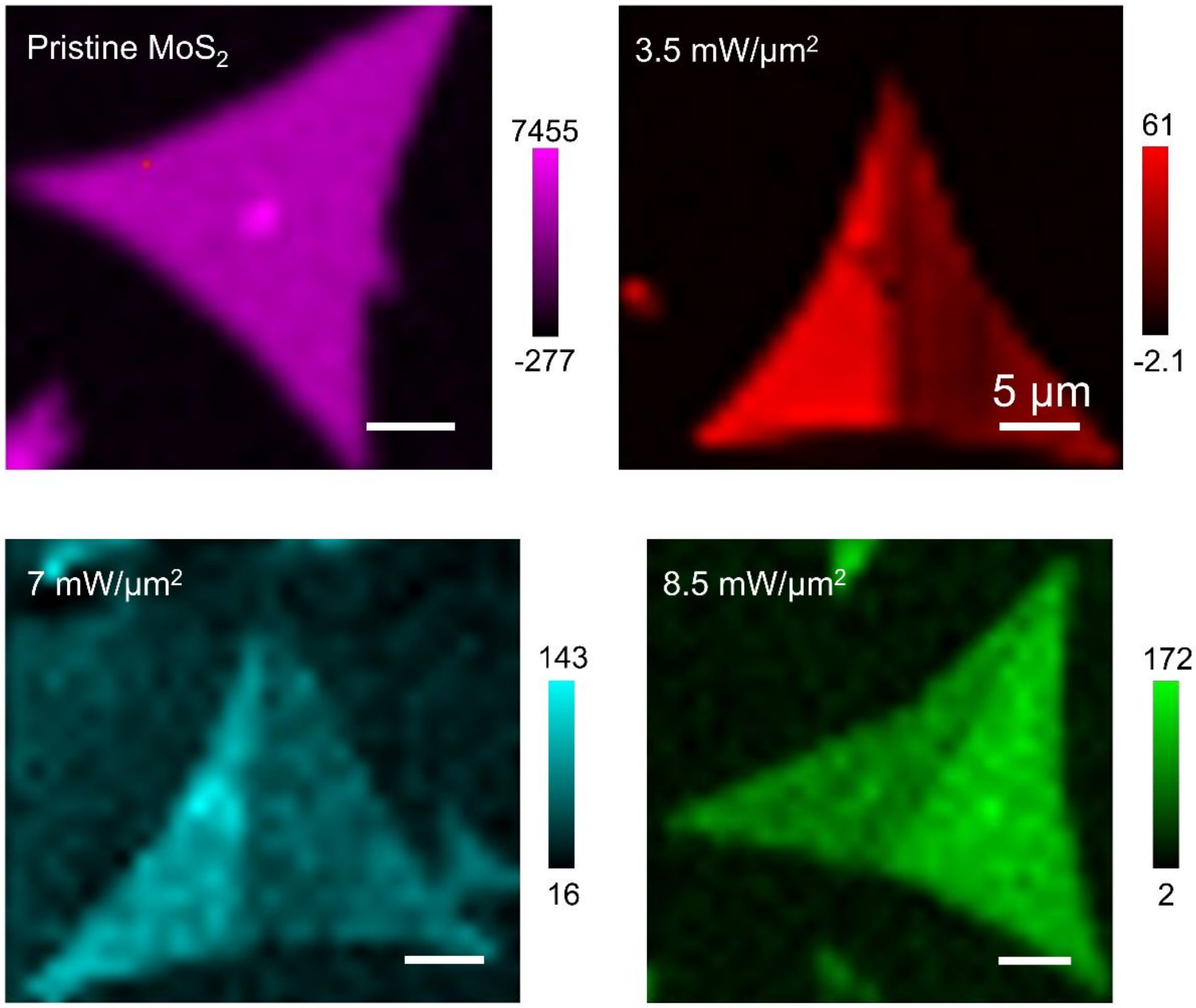


**Figure S7.** PL intensity (at 1.825 eV) mapping images of pristine $MoS_2$ and laser patterned (3.5, 7, and 8.5 mW/μm$^2$ laser density) $MoS_2$. The PL intensity at ~1.825 eV diminishes with increasing laser power. This quenching is attributed to S vacancies, which introduce deep defect bands between conduction band (CB) and valance band (VB) that act as efficient carrier trapping sites, promoting non-radiative decay and thereby reducing radiative recombination.[5-7] Additionally, the uniform intensity distribution across the patterned regions indicates a spatially uniform defect creation.

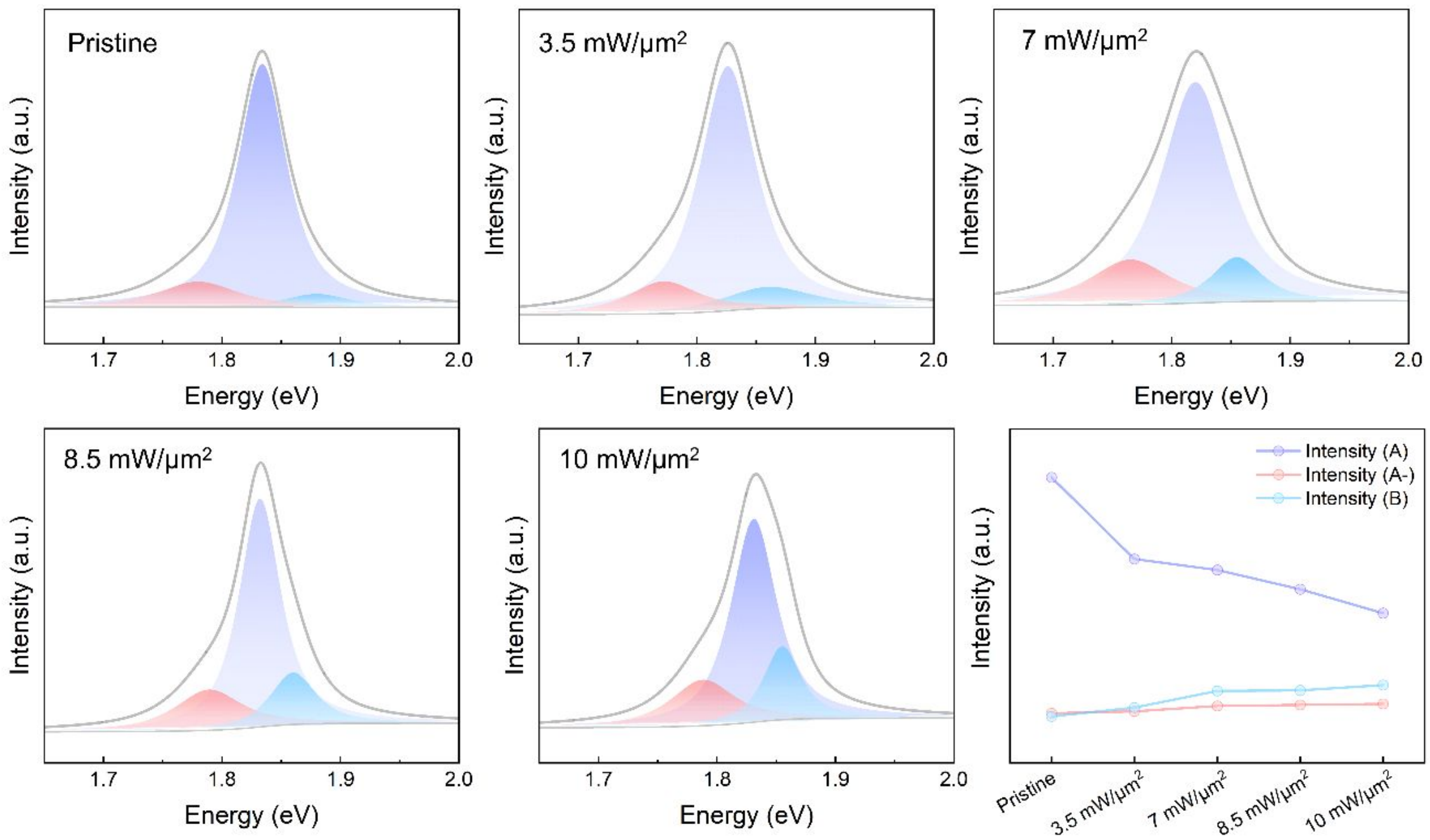


**Figure S8.** Photoluminescence (PL) analysis of excitonic emissions of $MoS_2$ before and after laser irradiation (3.5, 7, 8.5, and 10 mW/μm$^2$), and the corresponding intensity analysis. The peak positions of A, B, and $A^-$ excitons in PL spectra remain uniform. However, intensities analysis shows a decrease in A exciton intensity, accompanied by increases in $A^-$ trion and B exciton intensities following laser treatment. This spectral redistribution indicates n-type electron doping from S vacancies, which supplies free carriers that convert neutral A excitons into negatively charged $A^-$ trions and alters carrier relaxation dynamics to favor B exciton radiative recombination.

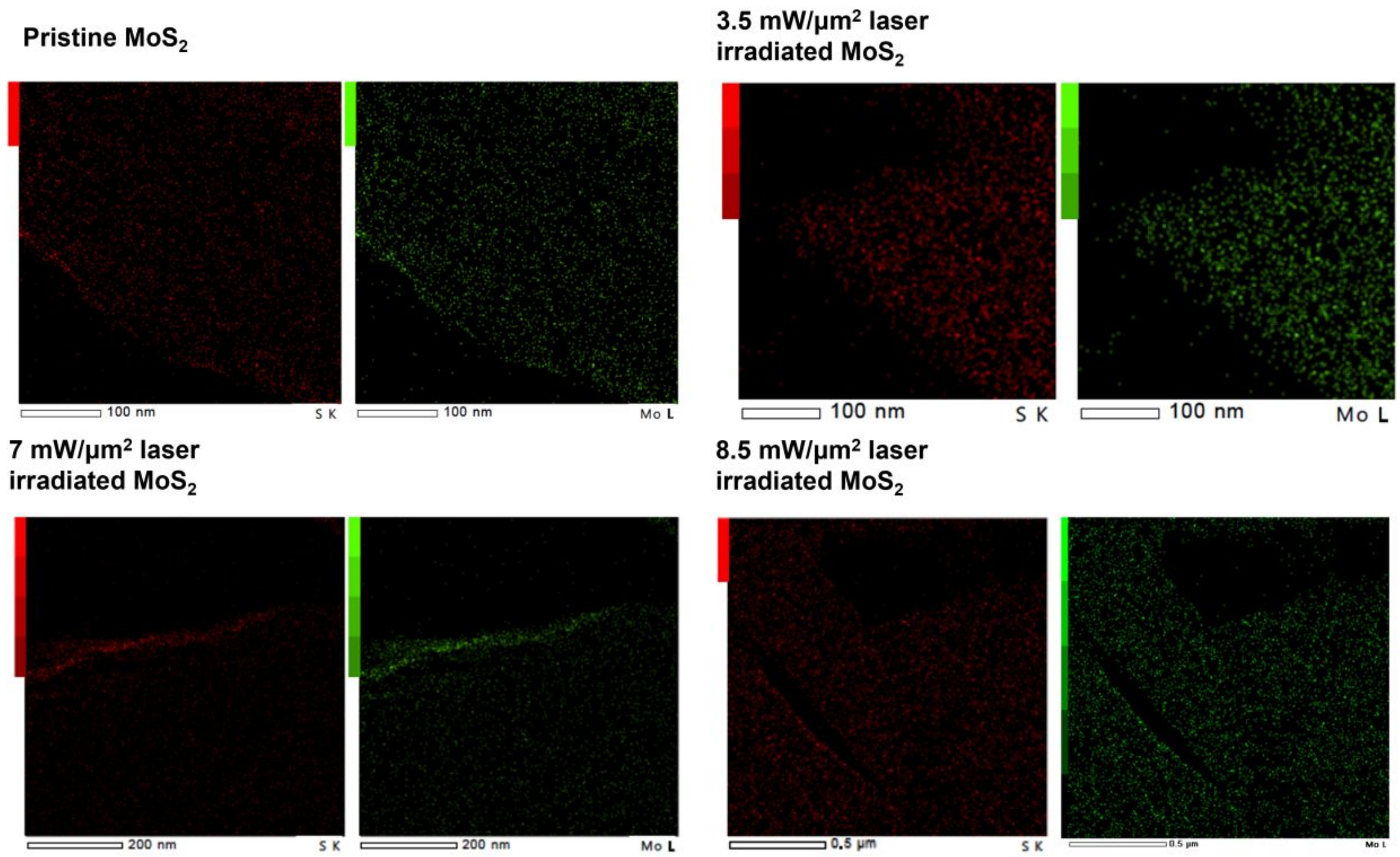


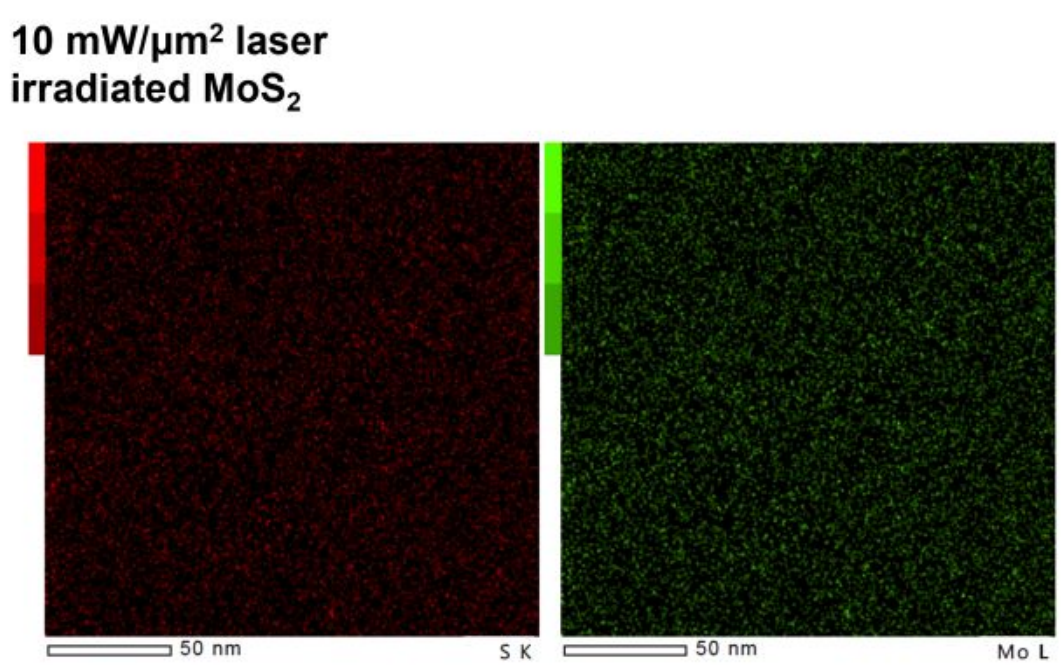


| Samples | Atomic percentage (at%) | |
|---|---|---|
| | Mo at% | S at% |
| Pristine $MoS_2$ | 62.13 | 37.87 |
| 3.5 mW/μm$^2$ | 63.31 | 36.69 |
| 7 mW/μm$^2$ | 64.19 | 35.81 |
| 8.5 mW/μm$^2$ | 65.20 | 34.80 |
| 10 mW/μm$^2$ | 65.78 | 34.22 |

**Figure S9.** Transmission electron microscopy-energy dispersive X-ray spectroscopy (TEM-EDS) images of pristine, 3.5, 7, 8.5 and 10 mW/μm$^2$ laser patterned $MoS_2$. The atomic ratio of Mo and S indicate that the increasing patterning laser density results in a reduction in the S atomic percentage, confirming the deliberate introduction of S vacancies with increasing density. The result shows that the S atomic percentages decrease with increasing laser patterning densities, confirming the controlled introduction of S vacancies in $MoS_2$.

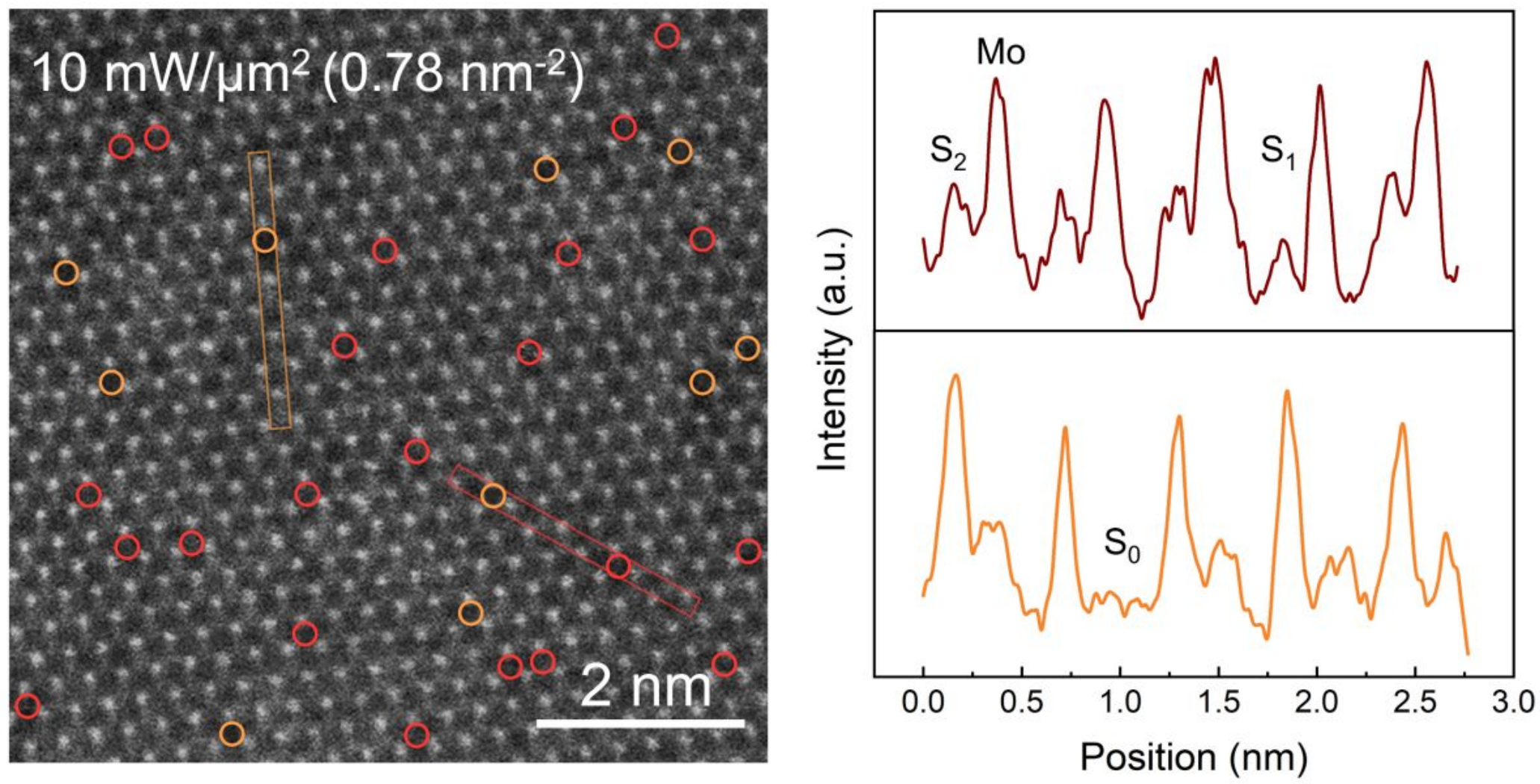


**Figure S10.** Line-profile analysis for the STEM image illustrated in **Fig. 2c** (left) and the corresponding line-profiles (right) reveal S vacancy (red) and di-S vacancy (orange) sites highlighted in the STEM image. Three distinct intensities are identified and correspond to Mo site (one Mo atom), $S_2$ site (two S atoms), $S_1$ site (one S atom is missing), and $S_0$ site (two S atom is missing), respectively. The analysis indicates that S vacancies are spatially isolated and not associated with any neighboring S vacancy site, consistent with the Density functional theory (DFT) model.

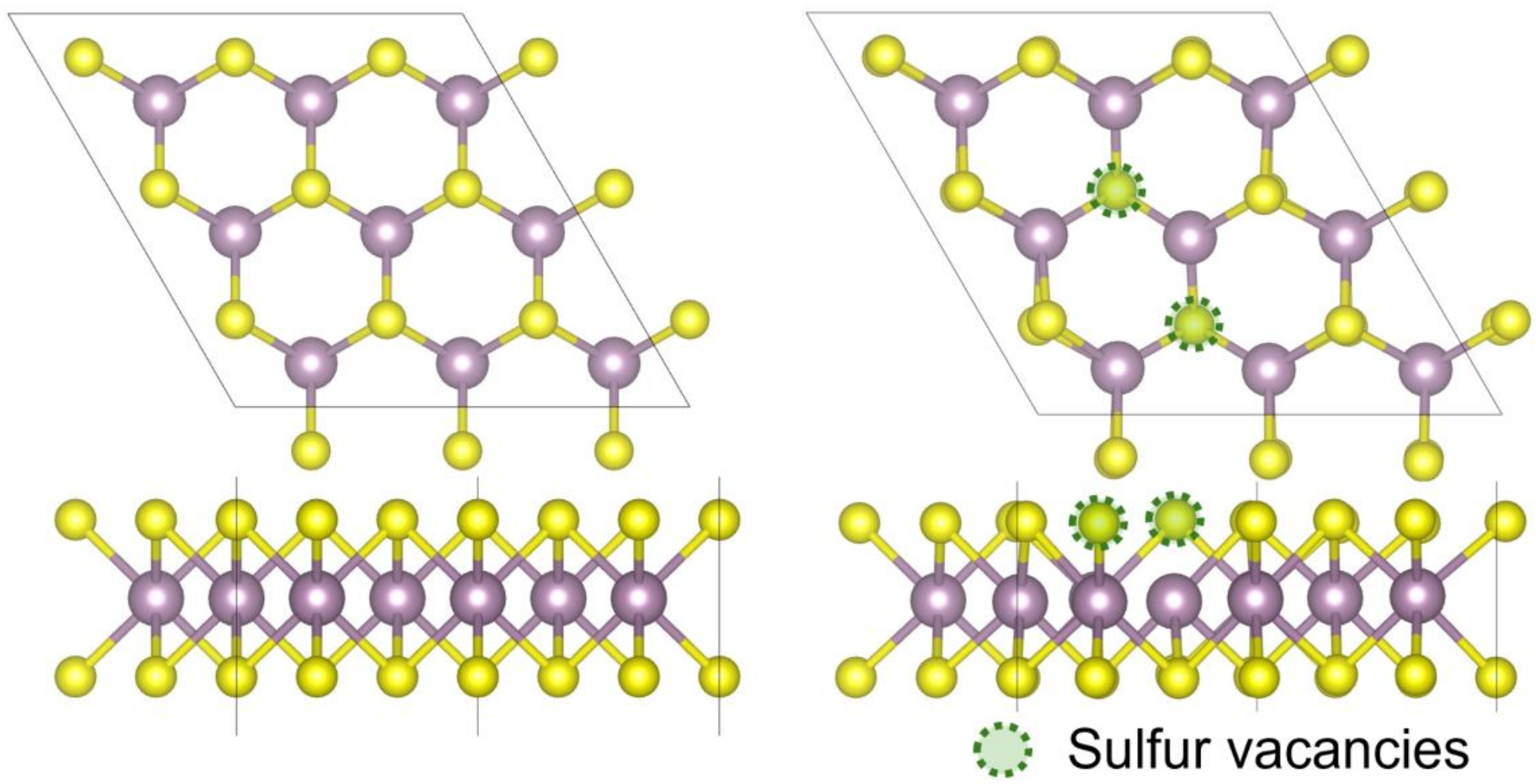


**Figure S11.** Density finctional theory (DFT)-optimized ground-state atomic structures of pristine $MoS_2$ and S vacancy $MoS_2$ with two S atoms removed from the lattice. With a quantified S vacancy density of only 0.06 nm$^{-2}$ (**Figures. S4**), the unirradiated $MoS_2$ exhibits exceptionally high crystalline quality and negligible electronic structure perturbation the $MoS_2$ without laser irradiation can be considered pristine and of high crystalline quality. This exceptionally low density is negligible in influencing the average electronic structure, thereby validating the use of an ideal lattice as a reasonable approximation in the DFT calculations

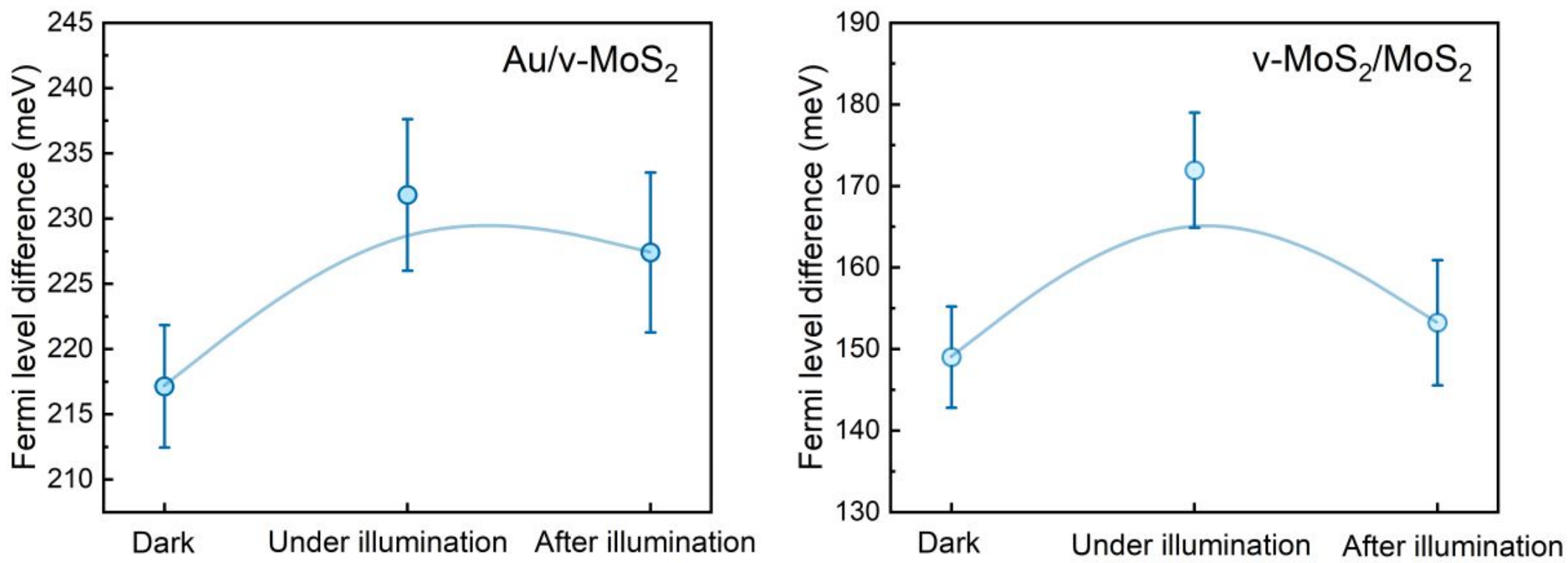


**Figure S12.** Line profile analysis of KPFM results from 20 datasets of surface potential differences between Au/v-$MoS_2$ and v-$MoS_2$/$MoS_2$ in the dark, under illumination, and after illumination. The consistent average values and small error bars across the 20 datasets confirm the uniformity of the built-in potential at the homojunction and reveal that the S vacancies serve as effective trapping sites for photogenerated carriers.

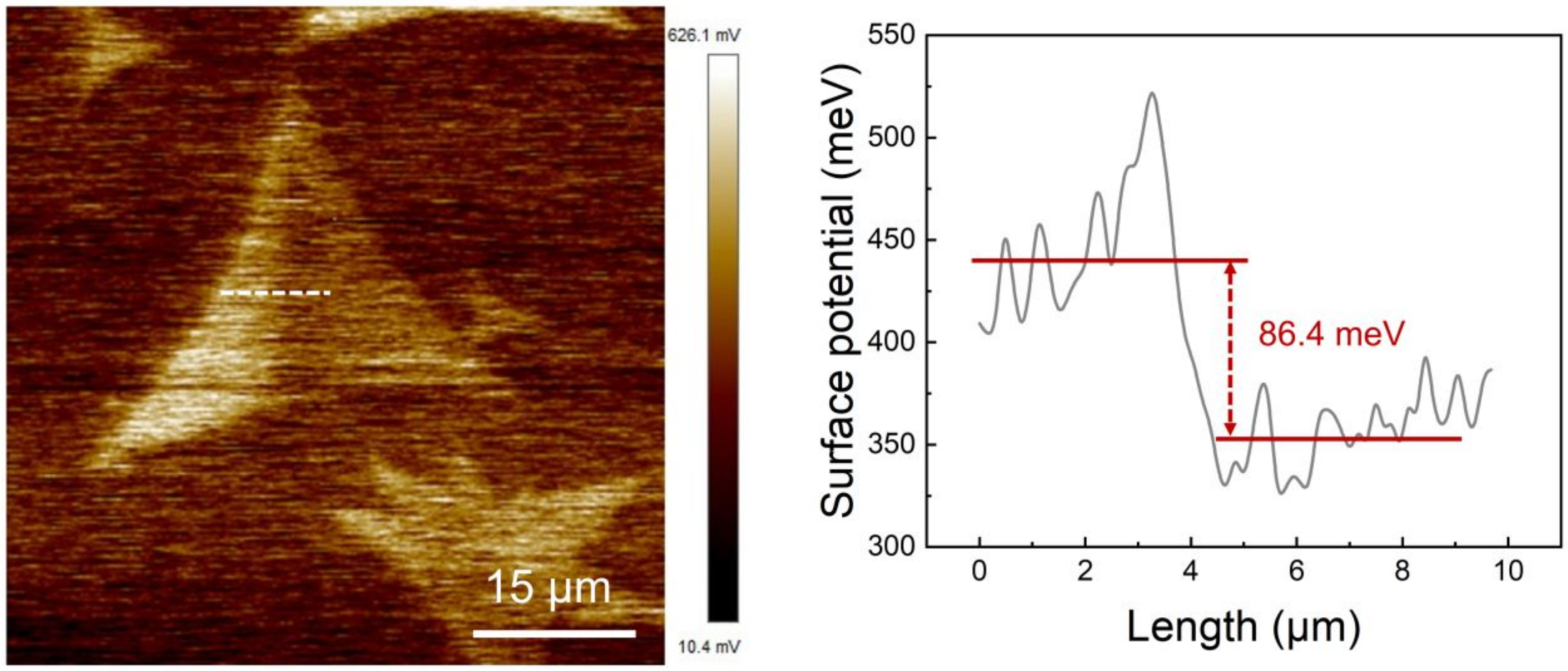


**Figure S13.** KPFM image of the homojunction between pristine $MoS_2$ and $MoS_2$ with 0.15 $nm^{-2}$ S vacancies (patterned at 3.5 mW/$\mu m^2$) under dark condition. The results show a smaller Fermi level (86.4 meV) compared to the homojunction of pristine $MoS_2$ and $MoS_2$ with 0.78 $nm^{-2}$ S vacancies (173 meV). The reduction in defect density results in a decrease in the Fermi level difference across the homojunction, indicating a weakening of the built-in electric field. This result offers direct experimental evidence that the magnitudes of the self-bias can be precisely adjusted by controlling the S vacancy densities through the variations in laser densities.

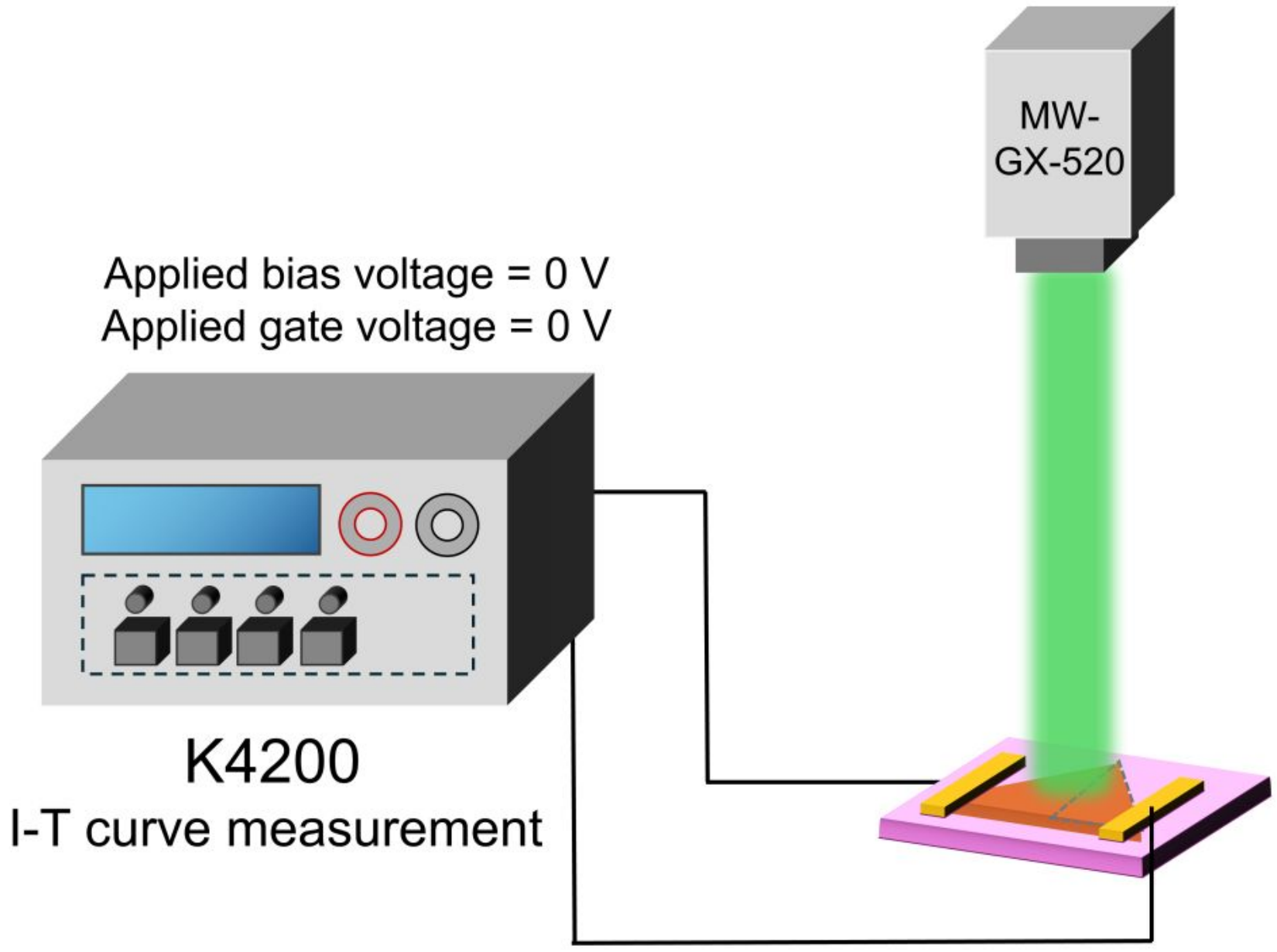


**Figure S14.** The illustration diagram of the postsynaptic current measurement configuration. A Keithley 4200 setup are used under zero gate voltage and source–drain bias (both $V_{gs}$ and $V_{ds}$ are set to 0 V). Additionally, no read voltage is used here, and the internal compliance current is set to $10^{-5}$ A. This further confirms that the measurable current observed under these conditions arises not from an applied bias but from the built-in electric field and asymmetric carrier trapping ability across the junction, which drives charge redistribution after optical stimulation. In addition, a relatively low light intensity (26.70 $mW/cm^2$) was used during all synaptic measurements to avoid introducing additional S vacancies, which is million times lower than the light intensity used for the S vacancies introduction (3.5 to 10 $mW/\mu m^2$). This ensures that the defect density remains constant throughout characterization, allowing the observed synaptic behavior to be attributed solely to the pre-treated S vacancy configurations. The relatively low photocurrent in our self-biased $MoS_2$ homojunction devices arises from two intrinsic factors. First, zero-external-bias operation limits the driving force for charge separation. Second, electron trapping-induced depletion of free charge carriers occurs as photogenerated electrons are intentionally captured at S vacancy sites, reducing the population of free electrons available for photocurrent.

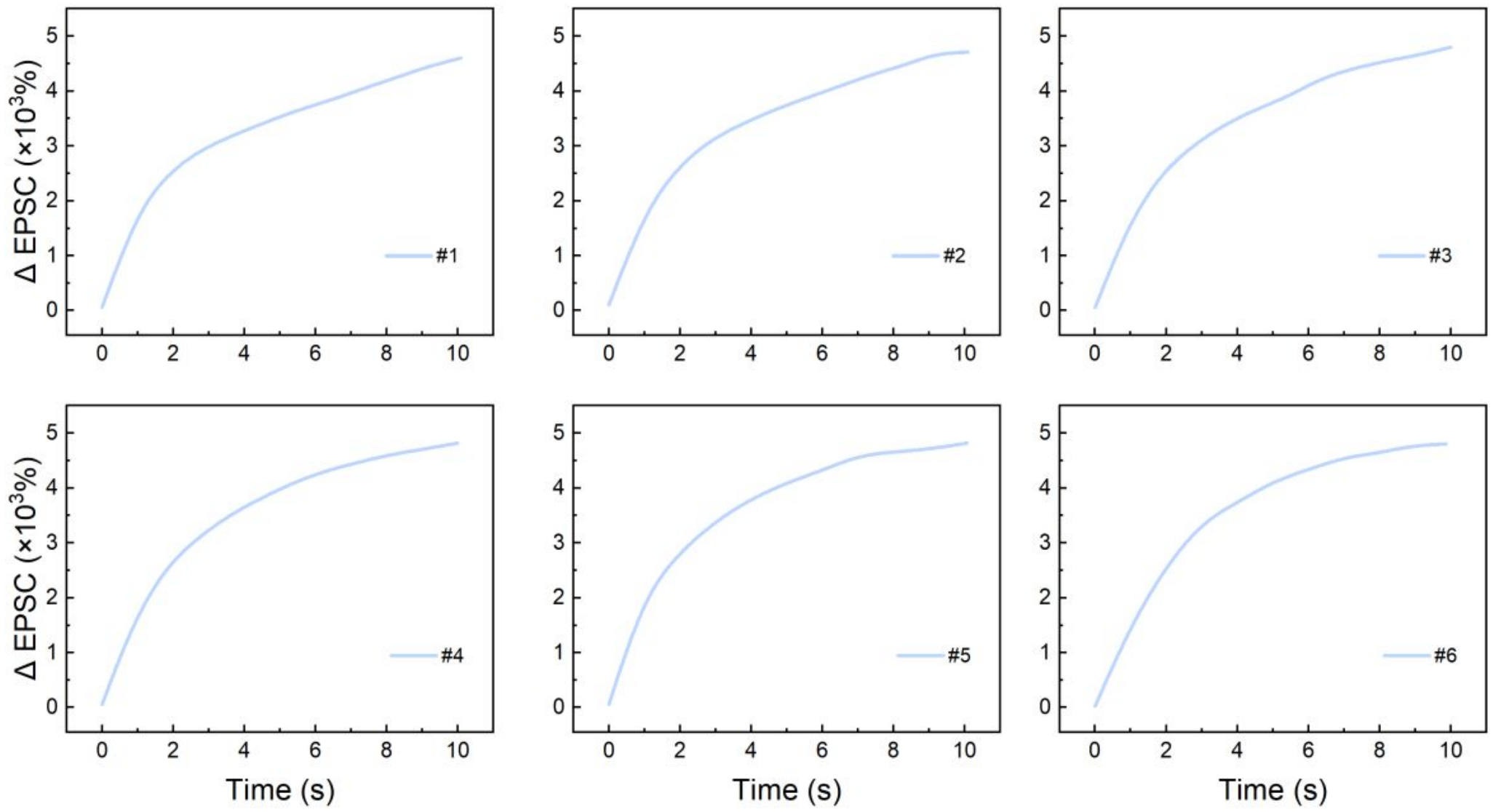


**Figure S15.** Uniformity of synaptic excitatory postsynaptic current (ΔEPSC) measured under 10 s optical exposure across multiple homojunction $MoS_2$ devices with an S vacancy density of 0.78 $nm^{-2}$. The uniformity of the postsynaptic current under 10 s optical exposure across multiple homojunction $MoS_2$ devices with an S vacancy density of 0.78 $nm^{-2}$ demonstrated excellent consistency and device-to-device reliability.

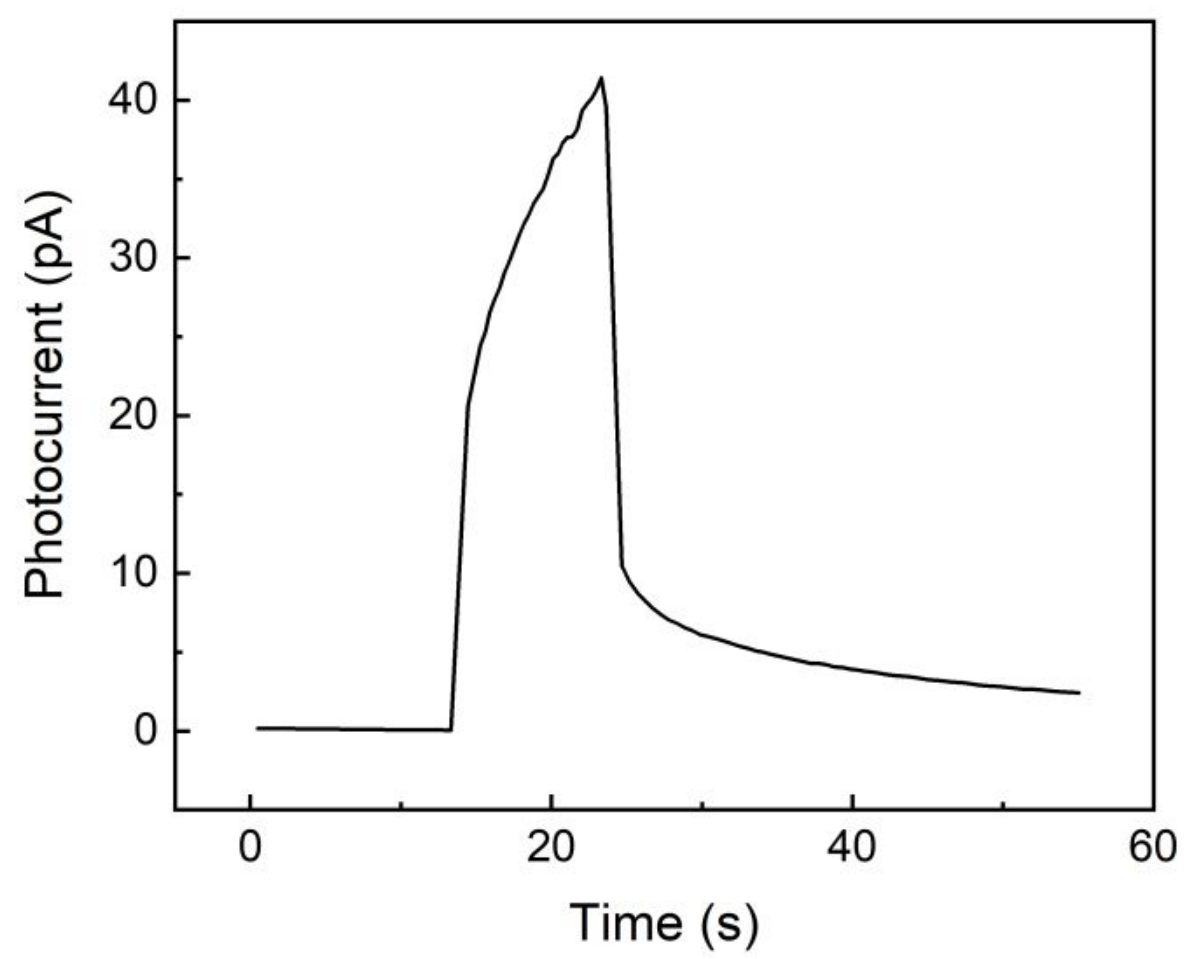


**Figure S16.** The photoresponse of the device shown in **Figure 4d** (laser patterned S vacancies $MoS_2$ sample with S vacancies density 0.78 $nm^{-2}$) after 50 days of storage under vacuum. The performance exhibited essentially the same postsynaptic current level as initially recorded, demonstrating that the S vacancy-induced homojunction device is stable over time.

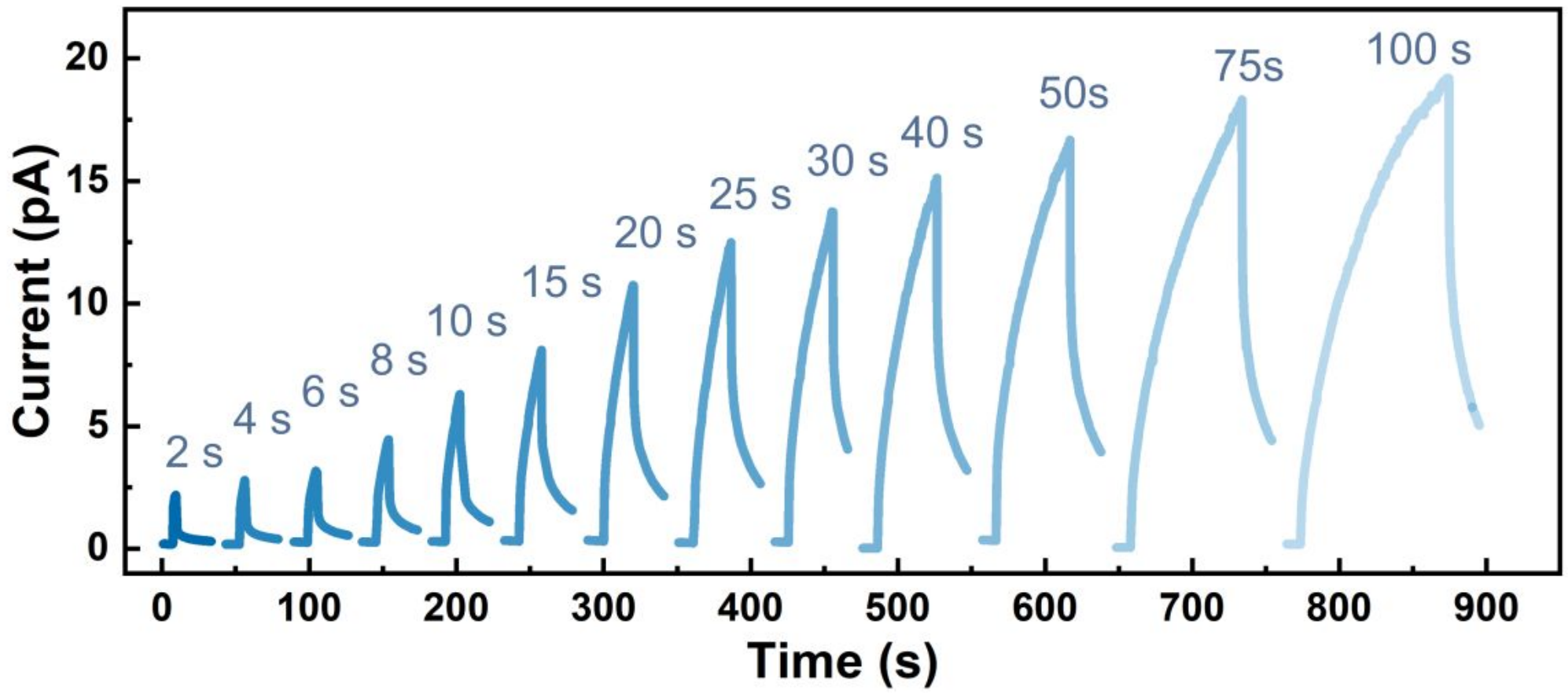


**Figure S17.** Exposure time-dependent photoresponse of the spatially laser-patterned $MoS_2$ with 0.78 $nm^{-2}$ S vacancies/pristine $MoS_2$ homojunction device. Extended illumination results in enhanced concentrations of the photogenerated electron-hole pairs, and contributes to an increased amplitude of the EPSC, revealing a systematic enhancement in the EPSC amplitude with longer exposure time.

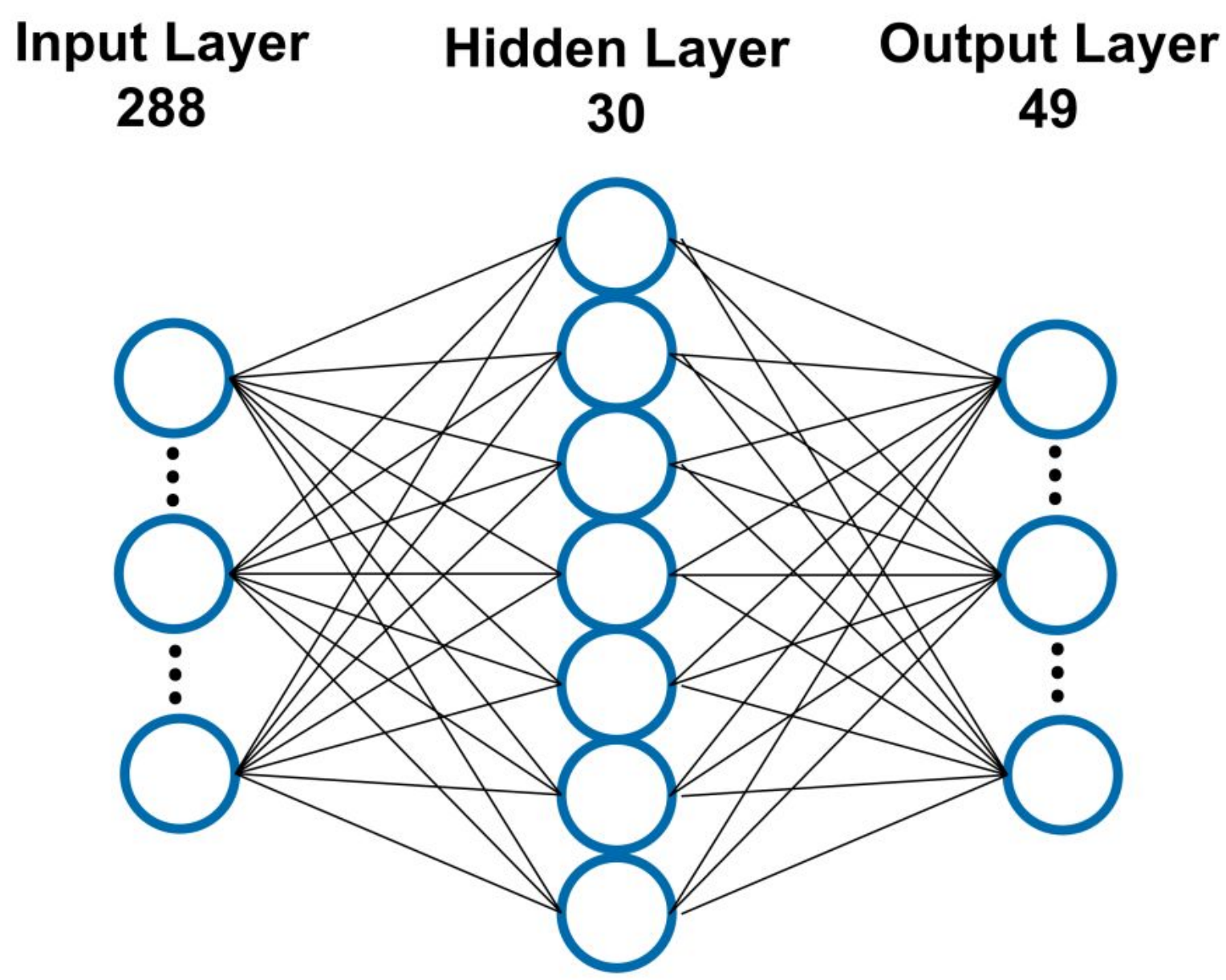


**Figure S18.** Schematic of the three-layer neural network. This architecture comprises three distinct layers: an input layer (288 nodes), a hidden layer (30 nodes), and an output layer (49 nodes), to execute the image recognition task.

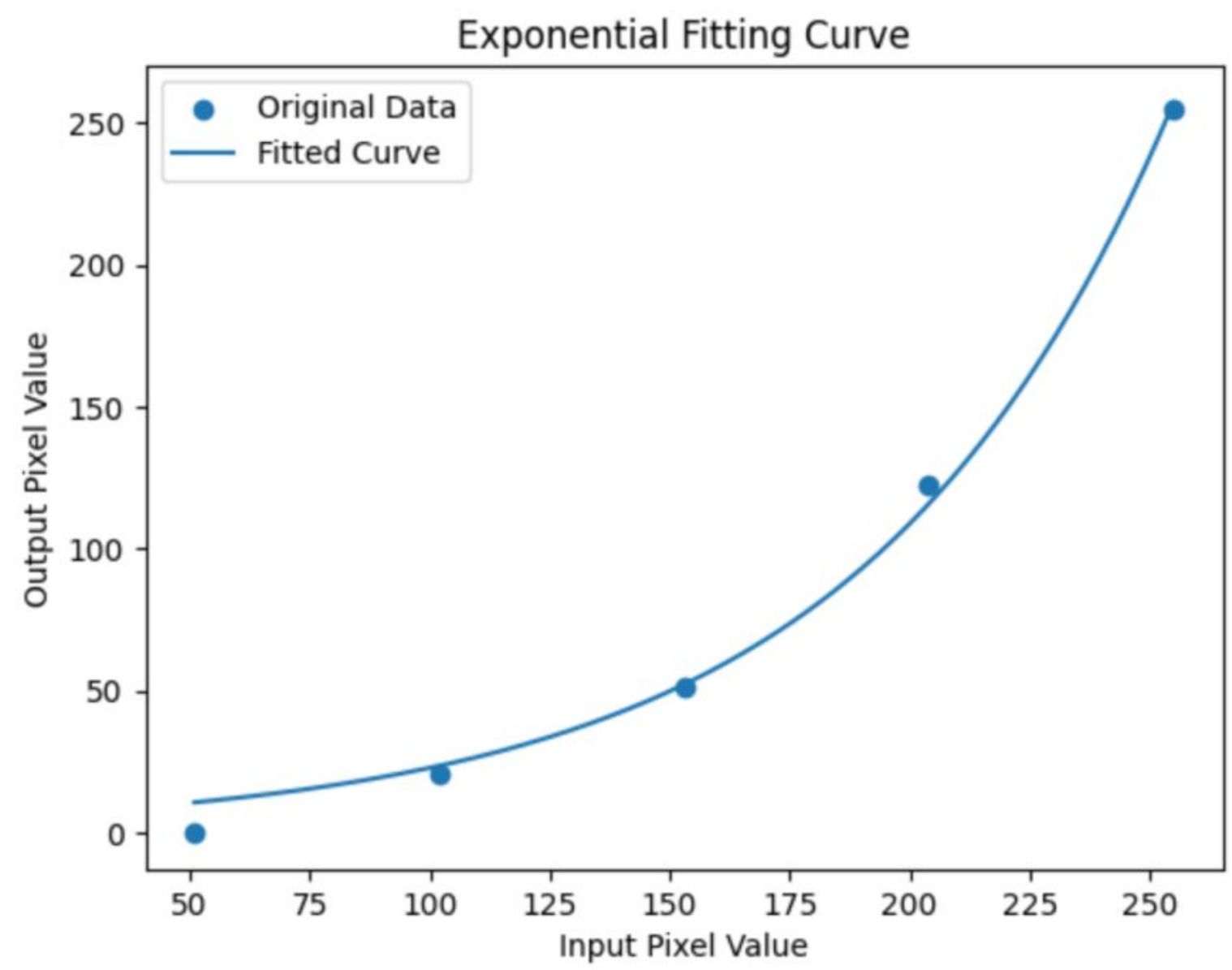


**Figure S19.** Exponential curve fitting of the denoising process in the neural network, with the relationship between synaptic preprocessing intensity and signal-to-noise ratio enhancement for motion-blurred license plate images. With the increase of optical exposure time, a significant enhancement in the postsynaptic current has been observed, enabling effective suppression of noise in signals.

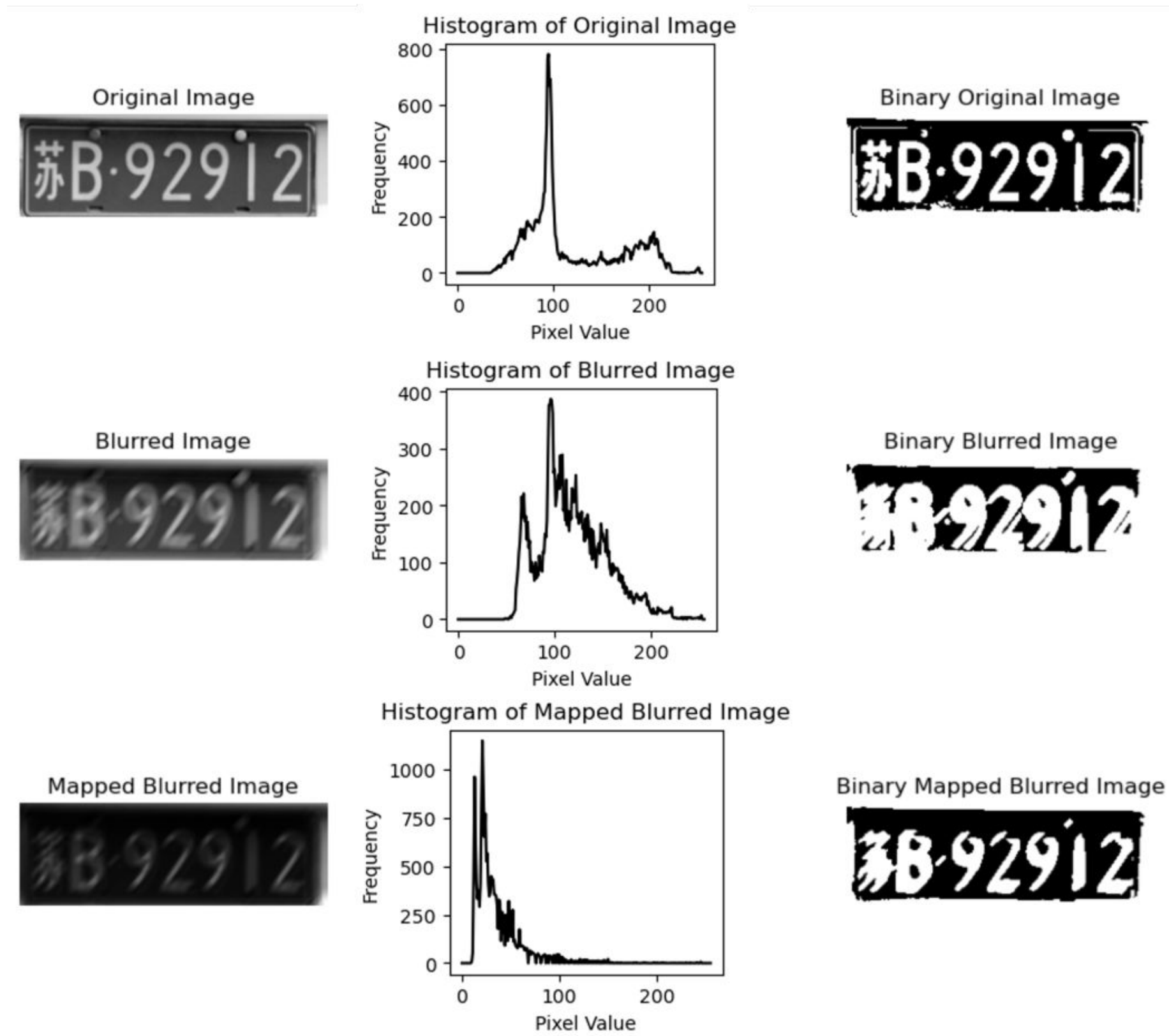


**Figure S20.** Grayscale value distribution statistics and corresponding binary images for clear, motion-blurred, and synaptically preprocessed license plates. The image comprising grayscale arrays with 288-pixel resolution has been implemented for training and validation within the artificial neuromorphic visual processing pipeline. The denoised images are statistically normalized, followed by binarization segmentation of characters and subsequently propagation through a fully connected neural network. A significant reduction in the statistical count is noted around the grayscale range of 70-150.

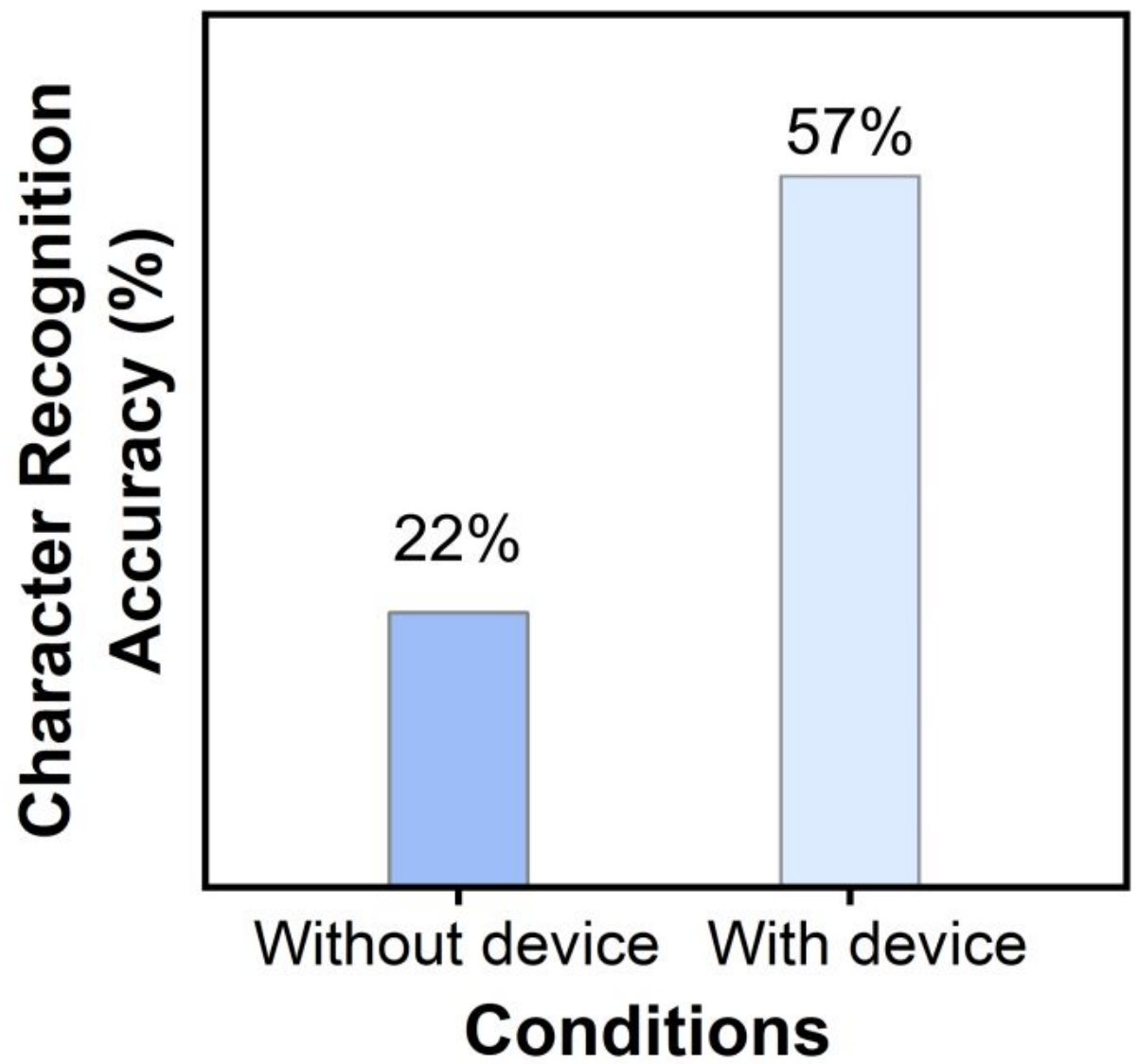


**Figure S21.** The corresponding character recognition accuracy improvement from 22% (without synaptic preprocessing) to 57% (with synaptic preprocessing). Leveraging the distinctive neuromorphic denoising capability, the signal-to-noise ratio for the motion-blurred license plate images is successfully enhanced through optically controlled synaptic facilitation. The process enabled clear segmentation of the alphanumeric characters, increasing character recognition accuracy from 22% to 57%.

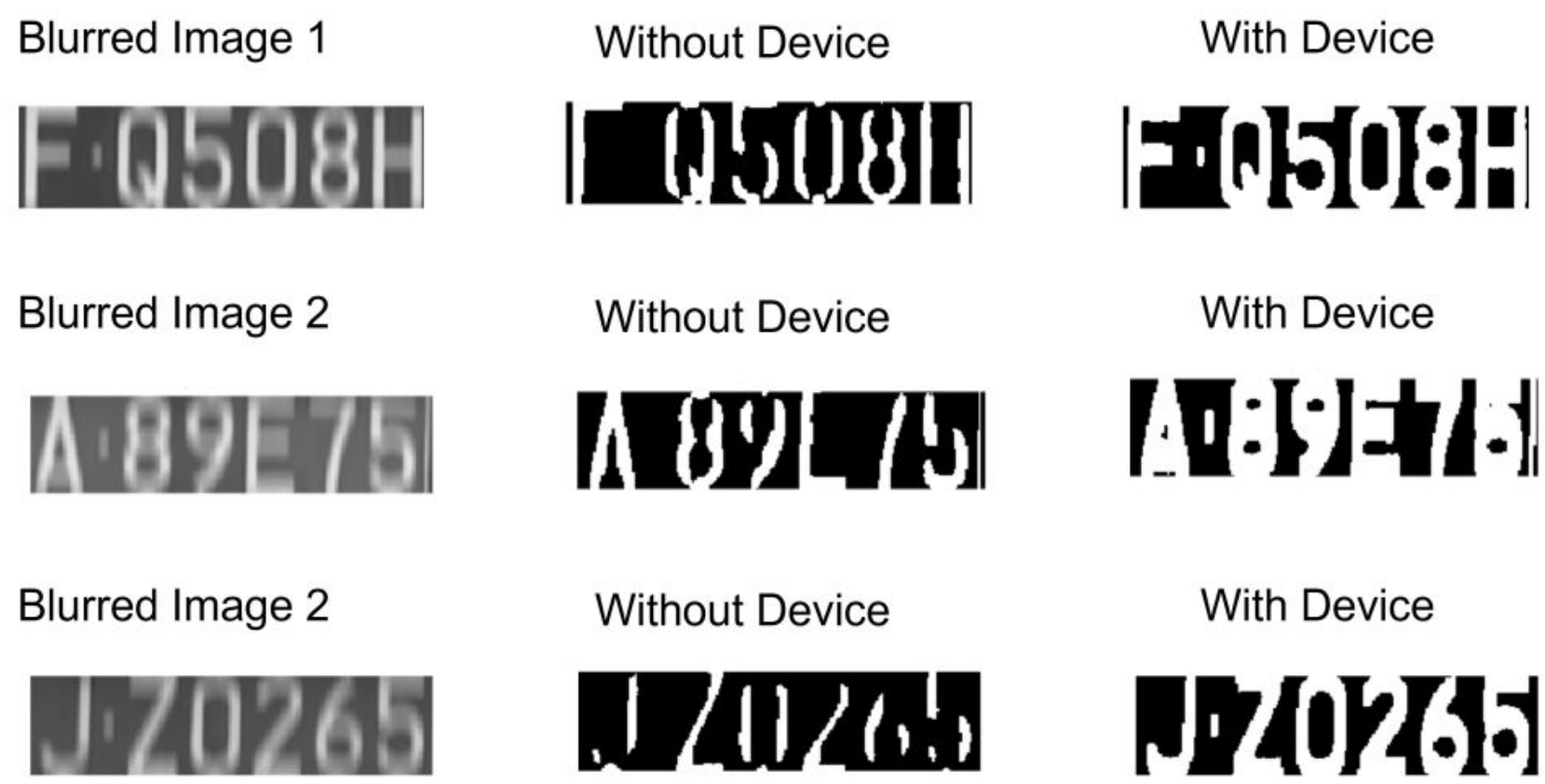


**Figure S22.** The motion-blurred license plate images before and after synaptic processing with enhanced feature clarity and significantly improved recognition accuracy.

**Polynomial fitting-based trajectory prediction**

The temporal sequence of target motion was reconstructed according to the variation order of photocurrent intensity in the simulated array output, followed by uniform trajectory sampling at fixed time intervals to generate historical position sequences. The results show that the conventional frame-based method, due to the limited number of available historical trajectory points, cannot accurately characterize complex motion trends, leading to significant deviations between predicted and actual trajectories. Moreover, when the polynomial order increases, the conventional method suffers from overfitting caused by insufficient data, resulting in larger prediction errors. In contrast, the optical synapse-based method benefits from high-density historical trajectory information, enabling stable fitting even with higher-order models.

Compared with conventional snapshot-based methods, which can only provide a limited number of historical points within the same observation window (set to three points in our experiments), the optical synaptic array provides much denser trajectory information, from which 21 historical position points were extracted in this work. Such high-density trajectory data provides more sufficient support for complex motion trajectory modeling. During the prediction stage, a polynomial fitting method based on historical position sequences was employed to estimate the future position of the target. To verify the effectiveness of the proposed approach, a dataset containing multiple motion trajectorie and conducted systematic evaluations is constructed. In addition, the prediction performance under different polynomial fitting orders was also investigated.

The trajectory prediction task aims to estimate the future position of a moving agent based on its historical motion. Let the trajectory be represented as a sequence of two-dimensional points:

$$\mathcal{T} = \{(x_i, y_i) | i = 0,1,\ldots,N-1\}ti,$$

where $(x_i, y_i)$ denotes the position at discrete time step $t = i$. The prediction problem can be formulated as estimating the future sequence:

$$\hat{\mathcal{T}} = \{(\hat{x}_j, \hat{y}_j) | j = N, N+1,\ldots,N+M-1\},$$

based on the observed past positions. A common approach is to approximate the evolution of $x_t$ and $y_t$ with polynomial regression models:

$$x(t) \approx f_x(t) = a_d t^d + a_{d-1} t^{d-1} + \cdots + a_1 t + a_0,$$
$$y(t) \approx f_y(t) = b_d t^d + b_{d-1} t^{d-1} + \cdots + b_1 t + b_0,$$

where $d$ is the polynomial degree. The coefficients $t\{a_i, b_i\}$ are obtained via least-squares fitting using the available historical data points. Predicted future positions are then derived by extrapolation:

$$(\hat{x}(t_f), \hat{y}(t_f)) = (f_x(t_f), f_y(t_f)),\ t_f \geq N.$$

**Table S1.** Prediction results under different polynomial orders

| Method | Second-order | Third-order | Fourth-order | Fifth-order |
|---|---|---|---|---|
| Device | 1.49 | 0.93 | 0.41 | 0.22 |
| w/o Device | 1.63 | 1.06 | 3.86 | 9.24 |

## References


(1) Yu, Z. G.; Zhang, Y.-W.; Yakobson, B. I. An Anomalous Formation Pathway for Dislocation-Sulfur Vacancy Complexes in Polycrystalline Monolayer MoS2. *Nano Letters* **2015**, *15*, 10, 6855-6861.

(2) Kang, T.; Lu, Z.; Liu, L.; et al. In Situ Defect Engineering of Controllable Carrier Types in WSe2 for Homomaterial Inverters and Self-Powered Photodetectors. *Nano Letters* **2023**, *23*, 23, 11034-11042.

(3) Tessarek, C.; Gridenco, O.; Wiesing, M.; et al. Controlled Laser-Thinning of MoS2 Nanolayers and Transformation to Amorphous MoOx for 2D Monolayer Fabrication. *ACS Applied Nano Materials* **2020**, *3*, 8, 7490-7498.

(4) Zhang, L.; Zhang, S.; Li, G.; et al. Ultrafast, one-step laser-induced in-situ transformation of MoS2 into α-MoO3 microribbons for high-performance triethylamine gas sensing. *Chemical Engineering Journal* **2026**, *530*, 173555.

(5) Li, Z.; Bretscher, H.; Zhang, Y.; et al. Mechanistic insight into the chemical treatments of monolayer transition metal disulfides for photoluminescence enhancement. *Nat Commun* **2021**, *12*, 1, 6044.

(6) Hong, J.; Hu, Z.; Probert, M.; et al. Exploring atomic defects in molybdenum disulphide monolayers. *Nat Commun* **2015**, *6*, 6293.

(7) Qiu, H.; Xu, T.; Wang, Z.; et al. Hopping transport through defect-induced localized states in molybdenum disulphide. *Nat Commun* **2013**, *4*, 2642.
